# The Alkaline Stability of Anion Exchange Membrane for Fuel Cell Applications: The Effects of Alkaline Media

**DOI:** 10.1002/advs.201800065

**Published:** 2018-06-04

**Authors:** Zhe Sun, Ji Pan, Jiangna Guo, Feng Yan

**Affiliations:** ^1^ State and Local Joint Engineering Laboratory for Novel Functional Polymeric Materials Department of Polymer Science and Engineering College of Chemistry Chemical Engineering and Materials Science Soochow University Suzhou 215123 China

**Keywords:** alkaline media, alkaline stability, anion exchange membranes, cationic polymers, density functional theory

## Abstract

Alkaline alcohols (methanol, ethanol, propanol, and ethylene glycol) have been applied as fuels for alkaline anion exchange membrane fuel cells. However, the effects of alkaline media on the stability of anion exchange membranes (AEMs) are still elusive. Here, a series of organic cations including quaternary ammonium, imidazolium, benzimidazolium, pyridinium, phosphonium, pyrrolidinium cations, and their corresponding cationic polymers are synthesized and systematically investigated with respect to their chemical stability in various alkaline media (water, methanol, ethanol, and dimethyl sulfoxide) by quantitative ^1^H nuclear magnetic resonance spectroscopy and density functional theory calculations. In the case of protic solvents (water, methanol, and ethanol), the lower dielectric constant of the alkaline media, the lower is the lowest unoccupied molecular orbital (LUMO) energy of the organic cation, which leads to the lower alkaline stability of cations. However, the hydrogen bonds between the anions and protic solvents weaken the effects of low dielectric constant of the alkaline media. The aprotic solvent accelerated the S_N_2 degradation reaction of “naked” organic cations. The results of this study suggest that both the chemical structure of organic cations and alkaline media (fuels) applied affect the alkaline stability of AEMs.

## 1 Introduction

Alkaline anion exchange membrane fuel cells (AEMFCs) in which the charge carrier is hydroxide anion instead of proton, are attracting significant interest due to their high power densities, enhanced oxygen reduction kinetics, high tolerances to CO_2_ in gaseous feeds, and the use of nonprecious electrocatalysts (such as cobalt, nickel, or silver).^1^, ^2^, ^3^, ^4^, ^5^, ^6^, ^7^, ^8^ Anion exchange membrane (AEM), which separates fuels and transports hydroxide anion between the anode and cathode, is one of the key components of AEMFCs. To fulfil a high power density of the fuel cell, an ideal AEM should possess high hydroxide conductivity, adequate chemical and mechanical stability in high pH solutions at elevated temperatures. Therefore, the development of AEMs with high alkali resistance stability has been one of the most important and challenging topics before the practical application and commercialization of AEMFCs.^1^, ^9^, ^10^

As a representative of AEM, quaternary ammonium based polymer membranes have been most extensively studied.^11^, ^12^, ^13^, ^14^, ^15^, ^16^, ^17^, ^18^, ^19^, ^20^, ^21^, ^22^, ^23^, ^24^ However, they are usually unstable in high pH solution, especially at high temperatures, probably due to the degradation via Hofmann elimination (E2),^25^ ylide formation (Y),^26^ and (or) nucleophilic substitution (S_N_2).^27^ To circumvent this obstacle and to achieve highly stable alkaline AEMs, alternative organic cations, including imidazolium,^28^, ^29^, ^30^, ^31^ guanidinium,^32^, ^33^ benzimidazolium,^34^, ^35^, ^36^ phosphonium,^37^, ^38^ pyrrolidinium,^39^, ^40^ pyridinium,^41^, ^42^ and metal cations^43^, ^44^ based AEMs have been recently synthesized and studied by experimental and (or) computational studies. Some of these AEMs show high alkaline stability and potential applications in AEMFCs. For example, Coates and co‐workers synthesized tetrakis‐(dialkylamino) phosphonium cation based polyethylene AEMs using ring‐opening metathesis polymerization.^37^ No significant loss of conductivity was observed in 15 m KOH solution at 22 °C over the 20 weeks period. Holdcroft and co‐workers reported that shielding the C2 position of benzimidazolium ring is an effective method to hinder the nucleophilic attack by hydroxide.^34^, ^35^, ^36^ Yan and co‐workers further demonstrated that the substitutions at the C2 or N3 positions of imidazolium (or pyrrolidinium) could highly enhance alkaline stability of corresponding AEMs at elevated temperatures.^28^, ^29^, ^39^

Usually, the chemical stability of the AEMs could be investigated via the changes in conductivity, ion exchange capacity (IEC), mechanical properties, as well as chemical structure changes. These changes could be characterized by electrochemical impedance,^45^, ^46^ neutralization titration,^28^, ^31^, ^47^ tensile testing,^39^ or spectroscopic methods (quantitative NMR, UV–vis, or Fourier transform infrared (FTIR) spectroscopy),^26^, ^48^, ^49^ respectively. Based on the results reported, the alkaline stability of these organic cations and their analogous AEMs has been tested using different test methods under the various tested conditions (e.g., alkaline media, and test temperatures). Therefore, it is quite difficult to compare the stability data of the cations and their corresponding AEMs investigated.

During the past years, alkaline alcohol (methanol, ethanol, propanol, and ethylene glycol) media have been applied as fuels for AEMFCs.^50^, ^51^, ^52^, ^53^ However, the effects of alkaline media on the alkaline stability of AEMs are rarely investigated. In this work, a series of organic cations including quaternary ammonium, imidazolium, benzimidazolium, pyridinium, phosphonium, pyrrolidinium cations, and their corresponding cationic polymers were synthesized. The alkaline stability of organic cations was investigated by ^1^H NMR in various alkaline media (water, methanol, ethanol, and dimethyl sulfoxide [DMSO]), as well as by the density functional theory (DFT) calculations. The dielectric constant effect of alkaline media on the stability of cations and their analogous AEMs were discussed.

## 2 Results and Discussion

### 2.1 Alkaline Stability of Organic Cations in D_2_O Solution

**Scheme 1:**
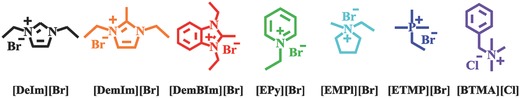
Molecular structures of cationic compounds investigated in this work.

**Scheme** **1** shows the chemical structure of the organic cations investigated in this work. The purity and chemical structures of these cationic compounds, including 1,3‐diethylimidazolium bromide ([DeIm][Br]), 1,3‐diethyl‐2‐methylimidazolium bromide ([DemIm][Br]), 1,3‐diethyl‐2‐methylbenzimidazolium bromide ([DemBIm][Br]), 1‐ethylpyridinium bromide ([EPy][Br]), 1‐ethyl‐1‐methylpyrrolidinium bromide ([EMPl][Br]), ethyltrimethylphosphonium chloride ([ETMP][Br]), benzyltrimethylammonium chloride ([BTMA][Cl]) were confirmed by ^1^H NMR (as shown in Figure S1 in the Supporting Information).

**Figure 1:**
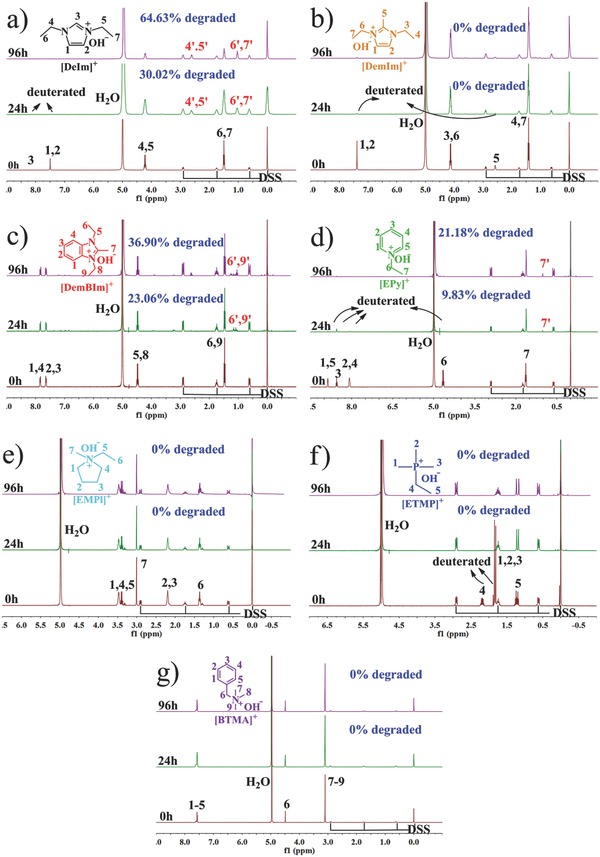
^1^H NMR spectra of a) [DeIm]^+^, b) [DemIm]^+^, c) [DemBIm]^+^, d) [EPy]^+^, e) [EMPl]^+^, f) [ETMP]^+^, and g) [BTMA]^+^ in 2 m KOH D_2_O solutions ([KOH]/[cation] = 15/1, molar ratio) at 80 °C for 24 and 96 h, respectively. DSS was used as an internal standard.

The alkaline stability of the synthesized cationic compounds was evaluated by quantitative ^1^H NMR spectroscopy. **Figure** **1** shows the ^1^H NMR spectra of the cationic compounds exposed in 2 m KOH D_2_O solution at 80 °C, using 3‐(trimethylsilyl)‐1‐propanesulfonic acid sodium salt (DSS) as an internal standard. The degradation degree of organic cations can be determined by calculating the relative integrated intensities of the indicated ^1^H resonances. It has been demonstrated the elevated temperature and high pH may facilitate the proton deuterium hydrogen/deuterium (H/D) exchange.^20^ After the treatment in 2 m KOH D_2_O solution at 80 °C for 24 h, the imidazolium, pyridinium, and phosphonium cations reacted quickly with D_2_O undergoing H/D exchange of the protons, and thus some signals disappeared. Such a H/D exchange was reversible in case of the solvent was changed back to H_2_O at 80 °C for 24 h, resulting in the reappearance of the corresponding proton signals (see Figure S2 in the Supporting Information).

Apart from H/D exchange, however, some new weak peaks appeared after being exposed to KOH solution at 80 °C for 96 h, especially for [DeIm]^+^, [DemBIm]^+^ and [EPy]^+^. The results indicate the degradation of organic cations in alkaline solutions. The degradation degree calculated by the relative integrated intensities was 64.63%, 34.90%, and 21.18% for [DeIm]^+^, [DemBIm]^+^, and [EPy]^+^, respectively (see Figure 1a,c,d). Therefore, the alkaline stability for the organic cations in 2 m KOH D_2_O solution was determined in the order: [EPy]^+^ > [DemBIm]^+^ > [DeIm]^+^. However, no degradation was observed for [DemIm]^+^, [EMPl]^+^, [ETMP]^+^, and [BTMA]^+^ under the same test conditions (see Figure 1b,e,f,g), implying a relatively higher alkaline stability. ^31^P NMR spectrum of the [ETMP]^+^ was further tested after the exposure in 2 m KOH D_2_O solution at 80 °C. It can be seen from Figure S4 in the Supporting Information that no new peaks were observed for [ETMP]^+^ even after168 h test, indicating high stability of [ETMP]^+^.

### 2.2 Alkaline Stability of Organic Cations in Various Alkaline Media (Methanol, Ethanol, and DMSO)

**Figure 2:**
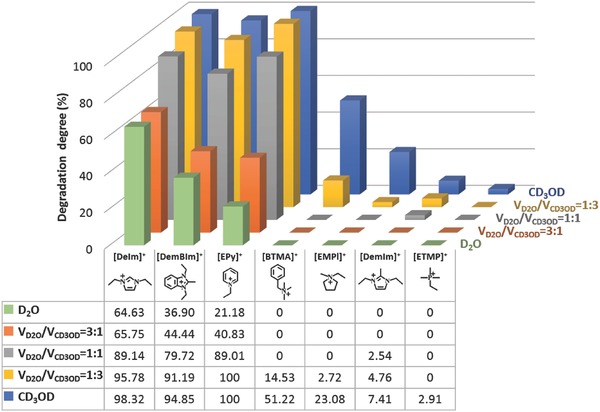
Alkaline degradation of organic cations in alkaline methanol solution (2 m KOH 80 °C, 96 h).

It has already been acknowledged that methanol is a clean and cheap fuel for alkaline fuel cell.^53^ To investigate the effect of methanol on the alkaline stability of organic cations, CD_3_OD was also applied as a co‐solvent for the NMR characterization. The synthesized organic cations were exposed in 2 m KOH solution (including $V_{D_{2}O}/V_{{\mathrm{CD}}_{3}\mathrm{OD}}=1:3$, $V_{D_{2}O}/V_{{\mathrm{CD}}_{3}\mathrm{OD}}=1:1$, $V_{D_{2}O}/V_{{\mathrm{CD}}_{3}\mathrm{OD}}=3:1$ and CD_3_OD) at 80 °C for 24 and 96 h, respectively (^1^H NMR spectra, see Figures S5–S8 in the Supporting Information). It can be seen that the addition of the methanol remarkably accelerated the alkaline degradation of organic cations. For instance, the degradation degree of [BTMA]^+^ in various alkaline media, including $V_{D_{2}O}/V_{{\mathrm{CD}}_{3}\mathrm{OD}}=3:1$, $V_{D_{2}O}/V_{{\mathrm{CD}}_{3}\mathrm{OD}}=1:1$, $V_{D_{2}O}/V_{{\mathrm{CD}}_{3}\mathrm{OD}}=1:3$ and CD_3_OD solution is about 0%, 0%, 14.53%, and 51.22%, respectively (see Figures S5g, S6g, S7g, and S8g, respectively in the Supporting Information). Moreover, both [ETMP]^+^ and [DemBIm]^+^ still showed relatively high alkaline stability even after the addition of methanol. Only about 7.41% of [DemBIm]^+^ and 2.91% of [ETMP]^+^ degraded in 2 m KOH CD_3_OD solution at 80 °C for 96 h, respectively (see Figure S8b,f, Supporting Information). In addition, the [DemBIm]^+^ and [ETMP]^+^ degraded about 87.01% and 48.98% in 4 m KOH CD_3_OD solution at 80 °C after 168 h test (see Figure S9 in the Supporting Information). The alkaline stability of the cations in CD_3_OD solution decreased in the order: [ETMP]^+^ > [DemIm]^+^ > [EMPl]^+^ > [BTMA]^+^ > [DemBIm]^+^ > [EPy]^+^ > [DeIm]^+^ (see **Figure** **2**).

**Figure 3:**
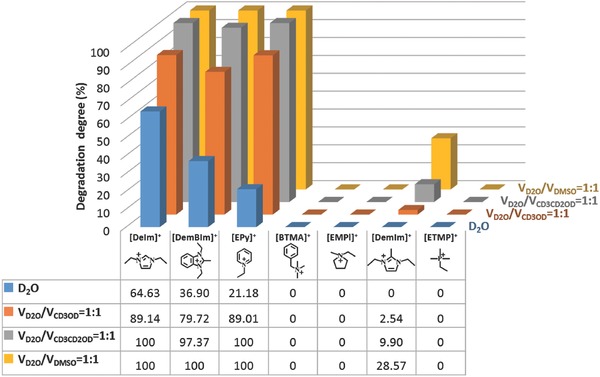
Alkaline degradation of various cations in $D_{2}O,V_{D_{2}O}/V_{{\mathrm{CD}}_{3}\mathrm{OD}}=1:1,\;\;\;V_{D_{2}O}/V_{{\mathrm{CD}}_{3}{\mathrm{CD}}_{2}\mathrm{OD}}=1:1\;\mathrm{and}\;V_{D_{2}O}/V_{\mathrm{DMSO}}=1:1\;\mathrm{alkaline}\;\mathrm{media}\;(2\;m\;\;\;\mathrm{KOH}\;\mathrm{80}\;{}^{\circ}C,\;\mathrm{96}\;h)$.

On the other hand, ethanol was also used as the co‐solvent for the alkaline stability test of organic cations. The ^1^H NMR spectra of synthesized organic cations exposed in 2 m KOH mixed solution $\left(V_{D_{2}O}/V_{{\mathrm{CD}}_{3}{\mathrm{CD}}_{2}\mathrm{OD}}=1:1\right)$ at 80 °C for 24 and 96 h are shown in Figure S10 in the Supporting Information. Compared with the alkaline methanol aqueous solution, similar accelerating effect for the degradation of organic cations was observed in alkaline ethanol solution (see **Figure** **3**). The degradation of the [DemIm]^+^, [BTMA]^+^ and [ETMP]^+^ was further studied in 2 m KOH ethanol solution $\left(V_{D_{2}O}/V_{{\mathrm{CD}}_{3}{\mathrm{CD}}_{2}\mathrm{OD}}=1:3\right)$ at 80 °C for 24 and 96 h, respectively. The degradation degree of [DemIm]^+^ is about 4.76% in $V_{D_{2}O}/V_{{\mathrm{CD}}_{3}\mathrm{OD}}=1:3$ solution, while more than 50.74% degraded in $V_{D_{2}O}/V_{{\mathrm{CD}}_{3}{\mathrm{CD}}_{2}\mathrm{OD}}=1:3$ solution under the same experimental condition (Figure S11a, Supporting Information). The same results were observed for [BTMA]^+^ and [ETMP]^+^ (see Figure S11b,c, Supporting Information).

To investigate the influences of aprotic solvent on the stability of organic cations, DMSO was chosen as the co‐solvent. Figure S12 in the Supporting Information shows the ^1^H NMR spectra of the synthesized organic cations exposed in 2 m KOH mixed solution $\left(V_{\mathrm{DMSO}}/V_{D_{2}O}=1:1\right)$ at 80 °C for 24 and 96 h, respectively. No significant degradation was observed for [EMPl]^+^, [ETMP]^+^, and [BTMA]^+^ in the mixed solution $\left(V_{\mathrm{DMSO}}/V_{D_{2}O}=1:1\right)$ (Figure S12e–g, Supporting Information). Compared with cations in D_2_O solution, the addition of DMSO highly accelerated the alkaline degradation of [DemIm]^+^, [DemBIm]^+^, and [EPy]^+^ (see Figure S12a,c,d in the Supporting Information). For example, the degradation degree of the [EPy]^+^ is about 21.18% in D_2_O solution (Figure 1d), while completely degraded (100%) in the mixed solution $\left(V_{\mathrm{DMSO}}/V_{D_{2}O}=1:1\right)$ after 24 h (Figure S12d, Supporting Information).

Figure 3 shows the alkaline degradation of various cations in water, methanol, ethanol, or DMSO alkaline solutions. As it can be seen, addition of methanol, ethanol, or DMSO significantly accelerated the degradation of the cations. The accelerating effect of alkaline media for the degradation of cations decreased in the order: DMSO > ethanol > methanol > water. For example, the degradation degree of [DemIm]^+^ was 0%, 2.54%, 9.90%, and 28.57% in D_2_O, $V_{D_{2}O}/V_{{\mathrm{CD}}_{2}\mathrm{OD}}=1:1,\;V_{D_{2}O}/V_{{\mathrm{CD}}_{3}{\mathrm{CD}}_{2}\mathrm{OD}}=1:1$, and $V_{\mathrm{DMSO}}/V_{D_{2}O}=1:1$ mixed solution, respectively.

As a consequence, it can be concluded that the alkaline stability of an organic cation is highly affected by both the chemical structure and the alkaline media. Choosing a suitable fuel is important for the applications of alkaline fuel cells.

### 2.3 DFT Calculations

**Figure 4:**
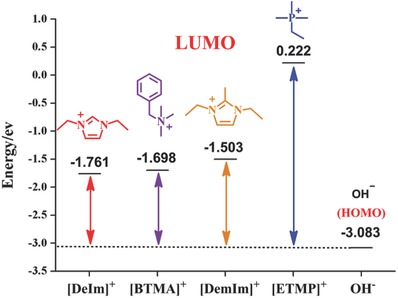
Frontier molecular orbital energy of various cations in water.

The organic cations, [DemIm]^+^, [BTMA]^+^, [DeIm]^+^, and [ETMP]^+^ were further studied by DFT calculations based on the molecular orbital theory. **Figure** **4** shows the lowest unoccupied molecular orbital (LUMO) energy of organic cations and the highest occupied molecular orbital (HOMO) energy of OH^−^ in D_2_O. As we can see that the LUMO energy of [DeIm]^+^, [BTMA]^+^, [DemIm]^+^, and [ETMP]^+^ in aqueous solution is determined to be −1.761, −1.698, −1.503 and 0.222 eV, respectively, while the HOMO energy of OH^−^ (in water) is calculated to be −3.083 eV, which is lower than the LUMO energy values of most of organic cations investigated in this work. It has been demonstrated that the nucleophilic attack of OH^−^ is restricted by the LUMO energy of the cations. The higher the LUMO energy, the more difficult it is for cations to be attacked by OH^−^ anions.^54^, ^55^, ^56^ Among the cations investigated above, the worst alkaline stability of [DeIm]^+^ may be due to its relatively lower LUMO energy (−1.761 eV). While the relatively higher LUMO energy of [ETMP]^+^ (0.222 eV) leads to a higher alkaline stability.

**Table** **1** shows the LUMO energy of organic cations in various alkaline media. As it can be seen the dielectric constant of solvent highly affected the LUMO energy of organic cations. The lower the dielectric constant of the solvent, the lower is the LUMO energy of organic cation, which leads to the lower alkaline stability of organic cations. The results could verify the experimental observations that the addition of methanol, ethanol, and DMSO accelerated the degradation of organic cations.

**Table 1:** LUMO energy of organic cations in various alkaline media

Cations	LUMO energy (eV)			
	Water (ε:78.54)	DMSO (ε:46.70)	Methanol (ε:32.63)	Ethanol (ε:24.30)
[DeIm]^+^	−1.761	−1.814	−1.870	−1.934
[BTMA]^+^	−1.698	−1.743	−1.783	−1.833
[DemIm]^+^	−1.503	−1.553	−1.606	−1.666
[ETMP]^+^	0.222	0.168	0.111	0.046
OH^−^(HOMO)	−3.083	−2.979	−2.869	−2.745

**Figure 5:**
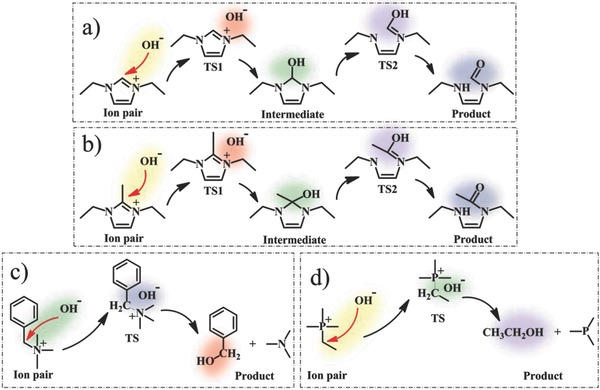
The S_N_2 degradation reaction process of a) [DeIm]^+^, b) [DemIm]^+^, c) [BTMA]^+^, and d) [ETMP]^+^.

It should be noted that the dielectric constant of DMSO (ε = 46.70) is higher than that of methanol (ε = 32.63) and ethanol (ε = 24.30), however, the worst alkaline stability of organic cations was observed in DMSO alkaline solution, if compared with those in methanol and ethanol. Therefore, the energy barrier (*E*_barrier_) values of cations were further calculated, which can be considered as an indispensable factor for the alkaline stability of cations. Here, *E*_barrier_ represents the energy that OH^−^ needed to overcome the energy difference between transition state (TS) and initial state and be used to evaluate the alkaline stability of cations attacked by OH^−^. **Figure** **5** shows the transition state and product that the [DeIm]^+^, [DemIm]^+^, [BTMA]^+^, and [ETMP]^+^ are attacked by OH^−^. The *E*_barrier_ values of [DeIm]^+^ and [DemIm]^+^ were studied only between the intermediate and initial state because the intermediate of both of them already lost the ability to deliver ions.

**Figure 6:**
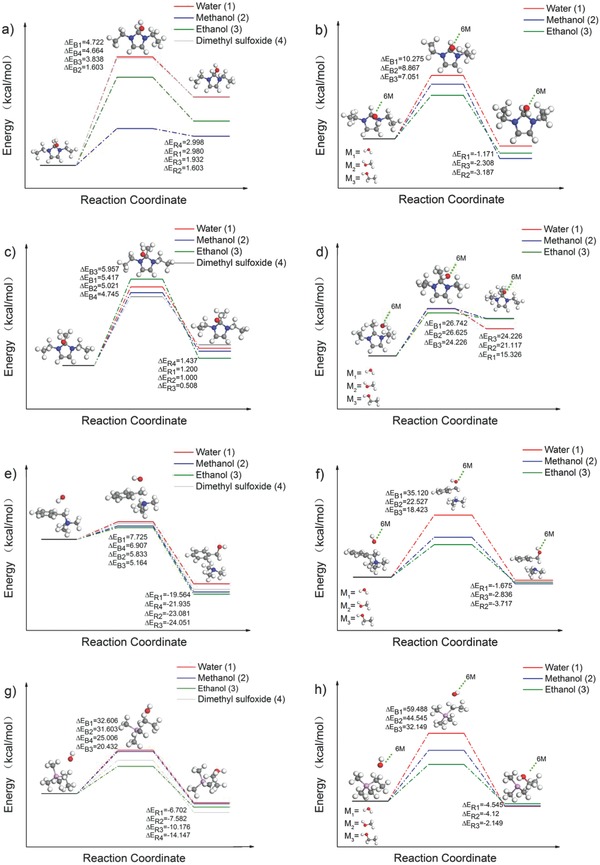
Reaction energy profiles for a,b) [DeIm]^+^, c,d) [DemIm]^+^, e,f) [BTMA]^+^, and g,h) [ETMP]^+^. Therein (b), (d), (f), and (h) was calculated through the way of introducing six protic solvent molecules to form the enveloping nucleophile (OH^−^) models. (*E*_B_: *E*_barrier_, *E*_R_: reaction energy, M: molecule).

It is worth noting that the hydrogen bonds can be formed between the nucleophile and the protic solvent (such as water, methanol, and ethanol), which would weaken the interactions between the nucleophile and the central atom. While in the aprotic solvent (DMSO), the negative charge of the anion is exposed in the solvent, as a result, such a “naked” anion has a high nucleophilicity. Therefore, the *E*_barrier_ value of cation in various solvents was simulated in two ways: 1) ignoring the influence of hydrogen bonds between the nucleophile and the protic solvent (water, methanol, ethanol) (see **Figure** **6**a,c,e,g); 2) emphasizing the effect of hydrogen bonds and introducing six protic solvent molecules to form enveloping nucleophile (OH^−^) models (see Figure 6b,d,f,h). Diesendruck and co‐workers reported the relationships between the number of water molecules solvating anion and the nucleophicility index of the hydroxide anion by DFT calculations.^24^ Here, the effects of alkaline media were mainly discussed by unifying the number of molecules per solvent. A great difference in the results of *E*_barrier_ values between the two calculation ways was obtained, especially for the *E*_barrier_ value of cations calculated through the establishment of an enveloping nucleophile model, which could rise more than threefold generally. For instance, the [DemIm]^+^ showed the lowest *E*_barrier_ values of 4.745 kcal mol^−1^ in DMSO solution, while the *E*_barrier_ value of [DemIm]^+^ could reach 26.74, 26.63, and 24.23 kcal mol^−1^ in water, methanol, and ethanol solution, respectively (see Figure 6c,d). The difference induced by protic or aprotic solvents may highly affect the alkaline stability of [DemIm]^+^, resulting in the worst alkaline stability of [DemIm]^+^ in DMSO solution. According to the *E*_barrier_ values presented in Figure 6, the organic cations showed diminishing *E*_barrier_ values in various alkaline media in the order of water, methanol, ethanol and DMSO. The order of which is opposite with the degradation degree of cations in these alkaline media. Meanwhile, the [ETMP]^+^ showed the highest *E*_barrier_ values in all the solvents. The theoretical calculation results were consistent with the experimental observations.

It is worth noting that both LUMO energy and *E*_barrier_ affected the stability of the cations. Although [BTMA]^+^ shows slightly higher LUMO energy than that of [DeIm]^+^ in water, the *E*_barrier_ of [BTMA]^+^ (35.120 kcal mol^−1^) is obviously higher than that of [DeIm]^+^ (10.275 kcal mol^−1^) (see Figure 6b,f), which improve the stability of [BTMA]^+^, much better than that of [DeIm]^+^. In the mixed solvent, the existence of two solvents may affect the *E*_barrier_ of the system and the stability of the cations simultaneously. Therefore, the [BTMA]^+^ shows higher stability than [DemIm]^+^ in the high water content solvent, while the [DemIm]^+^ is more stable than [BTMA]^+^ with the increasing amount of the co‐solvent methanol. The different *E*_barrier_ value of [DemIm]^+^ and [BTMA]^+^ determines the stability of [DemIm]^+^ and [BTMA]^+^ in the water and methanol, respectively (see Figure 6d,f).

### 2.4 Alkaline Stability of Cationic Polymers in Various Alkaline Media (Water, Methanol, and DMSO)

**Scheme 2:**
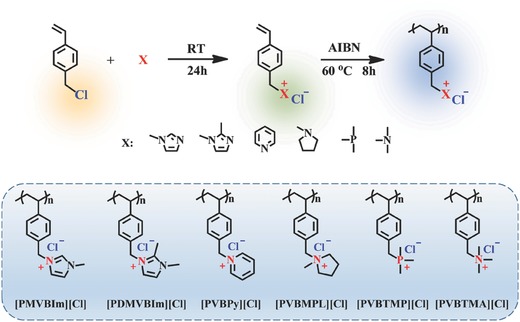
The synthesis routs of cationic homopolymers, and molecular structures of cationic homopolymers studied in this work.

Based on the alkaline stability results of the small molecule cations studied above, corresponding cationic polymers with pendant cations, including poly(1‐methyl‐3‐(4‐vinylbenzyl) imidazolium chloride) ([PMVBIm][Cl]), poly(1,2‐Dimethyl‐3‐(4‐vinylbenzyl)imidazolium chloride) ([PDMVBIm][Cl]), poly((4‐vinylbenzyl)pyridinium chloride) ([PVBPy][Cl]), poly(1‐(4‐vinylbenzyl)‐1‐methylpyrrolidinium chloride) ([PVBMPL][Cl]), poly((4‐vinylbenyl) trimethylphosphonium chloride) ([PVBTMP][Cl]), and poly((4‐vinylbenyl) trimethylammonium chloride) ([PVBTMA][Cl]) were synthesized via free radical polymerization (**Scheme** **2**). The chemical structures and purity of these cationic polymers were also confirmed by ^1^H NMR (Figure S14, Supporting Information). The IEC value of synthesized cationic polymers was calculated based on the monomer ratio (see Table S1 in the Supporting Information). The alkaline stability of the synthesized polymers was studied in 2 m KOH D_2_O/CD_3_OD mixed solutions $\left(D_{2}O,\;V_{D_{2}O}/V_{{\mathrm{CD}}_{3}\mathrm{OD}}=1:1,\;{\mathrm{CD}}_{3}\mathrm{OD}\right)$ at 80 °C for 24 and 96 h, respectively, as shown in Figures S15–S17 in the Supporting Information.

As it can be seen that [PVBMPL]^+^ and [PVBTMA]^+^ showed relatively high alkaline stability after being exposed in 2 m KOH D_2_O solution at 80 °C for 96 h (no significant degradation was observed), while the [PMVBIm]^+^ and [PVBPy]^+^ degraded completely after 24 h test (see Figure S15a,c in the Supporting Information). The relative alkaline stability order of these polymers is the same as that of the organic cations investigated, except for [PDMVBIm]^+^ (see Figure S15b in the Supporting Information). The possible reason might be due to the attached benzyl ring which can destroy the conjugated structure of imidazolium ring, making the electron cloud around cations more polarized, and leading to the easier attacking by OH^−^.^29^ Therefore, [PDMVBIm]^+^ showed worse stability compared with the corresponding small molecule cation [DemIm]^+^. It is not surprising that the addition of methanol can accelerate the degradation of cationic polymers (Figures S16 and S17, Supporting Information). For example, the [PVBTMA]^+^ which is quite stable (0% degradation degree) in 2 m KOH D_2_O solution (see Figure S15f in the Supporting Information), however, about 92.75% degraded under the same experimental condition in 2 m KOH CD_3_OD solution (see Figure S17f in the Supporting Information).

Hydrogen atoms substituted to phosphonium cation are quite active, therefore, both the H/D exchange and degradation of [PVBTMP]^+^ may occur simultaneously in alkaline solution (see Figures S15e, S16e, and S17e in the Supporting Information). Therefore, it was difficult to evaluate the degradation degree of [PVBTMP]^+^ only by ^1^H NMR spectra. The alkaline stability of [PVBTMP]^+^ in D_2_O, $V_{D_{2}O}/V_{{\mathrm{CD}}_{3}\mathrm{OD}}=1:1$ and CD_3_OD alkaline media was further characterized by ^31^P NMR (see Figures S18 in the Supporting Information). As it can be seen that [PVBTMP]^+^ degraded quickly in alkaline media, even at room temperature (see Figure S18 in the Supporting Information). The results of ^31^P NMR obviously demonstrated that [PVBTMP]^+^ was unstable in alkaline media.

To further understand the alkaline stability of [PVBTMP]^+^, the corresponding small cationic molecule, benzyltrimethylphosphonium ([BTMP]^+^), was synthesized and characterized by both ^1^H NMR (see Figure S19 in the Supporting Information) and ^31^P NMR spectra (see Figure S20 in the Supporting Information). Similar to [PVBTMP]^+^, very poor stability of [BTMP]^+^ in D_2_O and CD_3_OD alkaline media was observed. Meanwhile, addition of methanol remarkably accelerated the alkaline degradation of [BTMP]^+^ and [PVBTMP]^+^.

It should be noted that there is a tremendous difference between the alkaline stability of [ETMP]^+^ and [BTMP]^+^. The electron character of substituent groups adjacent to the cation site highly affected the alkaline stability of phosphonium cations. In the case of [ETMP]^+^, phosphonium was substituted with four alkyl chains (three methyl and one ethyl groups), while three methyl and one benzyl groups were adjacent to [BTMP]^+^. Such a different electronic environment may affect the alkaline stability of phosphonium cations. Since PhCH_2_^+^ is more stable than CH_3_CH_2_^+^, phosphonium cation with benzyl group ([BTMP]^+^) shows worse alkaline stability than fully alkyl chain substituted phosphonium ([ETMP]^+^).^57^, ^58^, ^59^, ^60^ Furthermore, the effect of benzyl groups on the alkaline stability of quaternary phosphonium is higher than that of quaternary ammonium cations, probably due to the longer bond length of C‐P (1.800 Å) than that of C‐N (1.499 Å).^60^, ^61^

**Figure 7:**
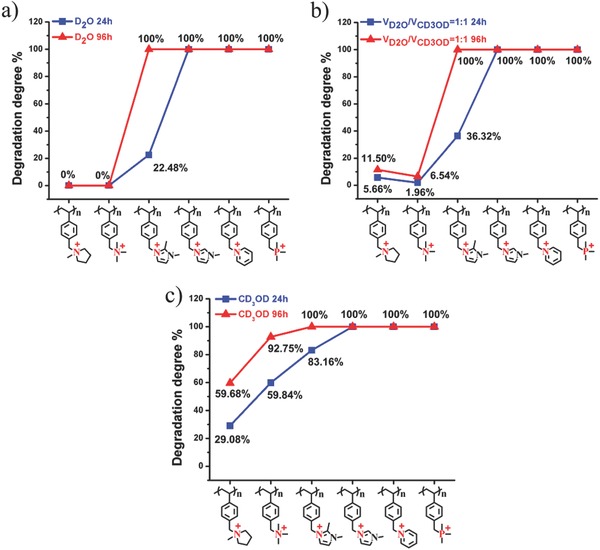
Degradation degree of cationic polymers under different test conditions: a) in 2 m KOH D_2_O solution at 80 °C for 24 and 96 h, respectively, b) in 2 m KOH mixed solution $\left(V_{D_{2}O}/V_{{\mathrm{CD}}_{3}\mathrm{OD}}=1:1\right)$ at 80 °C for 24 and 96 h, respectively, c) in 2 m KOH CD_3_OD solution at 80 °C for 24 and 96 h, respectively.

Among the cationic polymers studied in this work, the [PVBMPL]^+^ showed best alkaline stability. The alkaline stability of the cationic polymers was determined in the order: [PVBMPL]^+^ > [PVBTMA]^+^ > [PDMVBIm]^+^ > ([PMVBIm]^+^, [PVBPy]^+^ and [PVBTMP]^+^) (see **Figure** **7**).

Based on the results obtained above, alkaline stable [PVBMPL]^+^ and [PVBTMA]^+^ were further studied in DMSO solution (unfortunately, [PVBTMP]^+^ was insoluble in $V_{D_{2}O}/V_{\mathrm{DMSO}}=1:1$ solution). The degradation degree of [PVBMPL]^+^ and [PVBTMA]^+^ was determined to be 23.08% and 29.08%, respectively, in 2 m KOH $V_{D_{2}O}/V_{\mathrm{DMSO}}=1:1$ solution at 80 °C for 96 h (see Figure S21 in the Supporting Information). In contrast, only 11.50% of [PVBMPL]^+^ and 6.54% of [PVBTMA]^+^ degraded in 2 m KOH $V_{D_{2}O}/V_{{\mathrm{CD}}_{3}\mathrm{OD}}=1:1$ solution at 80 °C for 96 h, respectively. The results further confirmed that the presence of DMSO could highly decrease the alkaline stability of the cationic polymers. The IEC values of the cationic polymers in 2 m KOH alkaline media at 80 °C for 96 h were calculated based on the results of NMR spectra, and summarized in Table S1 in the Supporting Information.

## 3 Conclusions

In summary, a series of organic cations including quaternary ammonium, imidazolium, benzimidazolium, pyridinium, phosphonium, pyrrolidinium cations, and their analogous cationic polymers were synthesized. The alkaline stability of organic cations and cationic polymers in various alkaline media (water, methanol, ethanol, and DMSO) was characterized by quantitative ^1^H NMR spectra and DFT calculations. The alkaline stability of organic cations and their corresponding cationic polymers were affected by both the chemical structures and the alkaline media (fuels) applied. Addition of DMSO, methanol or ethanol as the co‐solvent (fuel) significantly accelerates the degradation of the organic cations. It is believed with the high development of the theoretical calculations and computational design, AEMs with long‐term stability and high performance could be developed and put into practical applications.

## 4 Experimental Section

*Materials*: Imidazole, 2‐methylimidazole, 2‐methylbenzimidazole, pyridine, 1‐methypyrroldine, trimethylphosphine tetrahydrofuran solution (1.0 mol L^−1^), trimethylamine (33% aqueous solution), ethyl bromide, acetonitrile, diethyl ether, 4‐vinylbenzyl chloride, 1‐methylimidazole, 1,2‐methylimidazole, (4‐vinylbenyl) trimethylammonium chloride, azobis(isobutyronitrile) (AIBN), DMSO, chloroform, potassium hydroxide, and 3‐(trimethylsilyl)‐1‐propanesulfonic acid sodium salt (DSS) were used as purchased. All the vinyl monomers were made inhibitor‐free by passing the liquid through a column filled with neutral Al_2_O_3_. Deionized water was used throughout the experiments.

*Synthesis of 1,3‐Diethylimidazolium Bromide ([DeIm][Br])*: [DeIm][Br] was synthesized by stirring a mixture containing imidazole (1.36 g, 0.02 mol), ethyl bromide (4.91 g, 0.045 mol), and KOH (1.12 g, 0.02 mol) in acetonitrile (30 mL) at room temperature under a nitrogen atmosphere for 48 h. The precipitate (KBr) was removed by filtration. After the evaporation of solvent, the mixture was washed three times with diethyl ether to obtain [DeIm][Br]. ^1^H NMR (400 MHz, D_2_O, δ): 8.745 (s, 1H; Im H), 7.463 (s, 2H; Im H), 4.209 (q, 4H; N‐CH_2_), 1.475 (t, 6H; CH_3_) (see Figure S1a in the Supporting Information).

*Synthesis of 1,3‐Diethyl‐2‐Methylimidazolium Bromide ([DemIm][Br])*: [DemIm][Br] was synthesized by stirring a mixture containing 2‐methylimidazole (1.64 g, 0.02 mol), ethyl bromide (4.91 g, 0.045 mol), and KOH (1.12 g, 0.02 mol) in acetonitrile (30 mL) at room temperature under a nitrogen atmosphere for 48 h. The precipitate (KBr) was removed by filtration. After the evaporation of solvent, the mixture was washed three times with diethyl ether to obtain ([DemIm][Br]). ^1^H NMR (400 MHz, D_2_O, δ): 7.355 (s, 2H; Im H), 4.104 (q, 4H; N‐CH_2_), 2.583 (s, 3H; CH_3_), 1.401 (t, 6H; CH_3_) (see Figure S1b in the Supporting Information).

*Synthesis of 1,3‐Diethyl‐2‐Methylbenzimidazolium Bromide ([DemBIm][Br])*: [DemBIm][Br] was synthesized by stirring a mixture containing 2‐methylbenzimidazole (2.64 g, 0.02 mol), ethyl bromide (4.91 g, 0.045 mol), and KOH (1.12 g, 0.02 mol) in acetonitrile (30 mL) at room temperature under a nitrogen atmosphere for 48 h. The precipitate (KBr) was removed by filtration. After the evaporation of solvent, the mixture was washed three times with diethyl ether to obtain [DemBIm][Br]. ^1^H NMR (400 MHz, D_2_O, δ): 7.824 (m, 2H; Ar H), 7.620 (m, 2H; Ar H), 4.475 (q, 4H; N‐CH_2_), 2.884 (s, 3H; CH_3_), 1.4960 (t, 6H; CH_3_) (see Figure S1c in the Supporting Information).

*Synthesis of 1‐Ethylpyridinium Bromide ([EPy][Br])*: [EPy][Br] was synthesized by stirring a mixture containing pyridine (1.58 g, 0.02 mol) and an equivalent molar amount of ethyl bromide (2.18 g, 0.02 mol) in diethyl ether (30 mL) at room temperature under nitrogen atmosphere for 48 h. After the evaporation of solvent, the mixture was washed three times with diethyl ether to obtain [EPy[Br]. ^1^H NMR (400 MHz, D_2_O, δ): 8.855 (d, 2H; Py H), 8.513 (t, 1H; Py H), 8.044 (t,2H; Py H), 4.644 (q, 2H; N‐CH_2_), 1.618 (t, 3H; CH_3_) (see Figure S1d in the Supporting Information).

*Synthesis of 1‐Ethyl‐1‐Methylpyrrolidinium Bromide ([EMPl][Br])*: [EMPl][Br] was synthesized by stirring a mixture containing 1‐methypyrroldine (1.70 g, 0.02 mol) and an equivalent molar amount of ethyl bromide (2.18 g, 0.02 mol) in diethyl ether (30 mL) at room temperature under nitrogen atmosphere for 48 h. After the evaporation of solvent, the mixture was washed three times with diethyl ether to obtain [EMPl][Br]. ^1^H NMR (400 MHz, D_2_O, δ): 3.480 (m, 4H; N‐CH_2_), 3.397 (q, 2H; N‐CH_2_), 3.017 (s, 3H; N‐CH_3_), 2.203 (m, 4H; CH_2_), 1.373 (t, 3H; CH_3_) (see Figure S1e in the Supporting Information).

*Synthesis of Ethyltrimethylphosphonium Bromide ([ETMP][Br])*: [ETMP][Br] was synthesized by stirring a mixture containing trimethylphosphine tetrahydrofuran solution (10 mL, 0.01 mol) and ethyl bromide (1.09 g, 0.01 mol) at room temperature under a nitrogen atmosphere for 48 h. After the evaporation of solvent, the mixture was washed three times with diethyl ether to obtain [ETMP][Br]. ^1^H NMR (400 MHz, D_2_O, δ): 2.187 (m, 2H; P‐CH_2_‐CH_3_), 1.832 (d, *J* = 14.0 Hz, 9H; P‐CH_3_), 1.223 (dt, *J* = 20.0, 8.0 Hz, 3H; P‐CH_2_‐CH_3_), ^31^P NMR (400 MHz, D_2_O, δ): 27.88. (see Figure S1f and Figure S3 in the Supporting Information).

*Synthesis of Benzyltrimethylammonium Chloride ([BTMA][Cl])*: [BTMA][Cl] was synthesized by stirring a mixture containing trimethylamine (33% aqueous solution) (3.94 g, 0.02 mol) and benzyl chloride (2.53 g, 0.02 mol) at room temperature under a nitrogen atmosphere for 48 h. After the evaporation of solvent, the mixture was washed three times with diethyl ether to obtain [BTMA][Cl]. ^1^H NMR (400 MHz, D_2_O, δ): 7.535 (m, 5H; Ar H), 4.471 (s, 2H; N‐CH_2_), 3.047 (s, 9H; N‐CH_3_) (see Figure S1g in the Supporting Information).

*Synthesis of Benzyltrimethylphosphonium Chloride ([BTMP][Cl])*: [BTMP][Cl] was synthesized by stirring a mixture containing trimethylphosphine tetrahydrofuran solution (10 mL, 0.01 mol) and benzyl chloride (1.27 g, 0.01 mol) at room temperature under a nitrogen atmosphere for 48 h. After the evaporation of solvent, the mixture was washed three times with diethyl ether to obtain [BTMP][Cl]. ^1^H NMR (400 MHz, D_2_O, δ): 7.450 (m, 3H; Ar H), 7.317 (m, 2H; Ar H), 3.674 (d, *J* = 15.6 Hz, 2H; P‐CH_2_), 1.800 (d, *J* = 14.4 Hz, 9H; P‐CH_3_) (see Figure S19 in the Supporting Information). ^31^P NMR (400 MHz, D_2_O, δ): 25.74 (see Figure S20a in the Supporting Information). ^31^P NMR (400 MHz, CD_3_OD, δ): 26.85 (see Figure S20b in the Supporting Information).

*Synthesis of 1‐Methyl‐3‐(4‐Vinylbenzyl)Imidazolium Chloride ([MVBIm][Cl])*: [MVBIm][Cl] was synthesized by stirring a mixture containing 1‐methylimidazole (1.64 g, 0.02 mol) and 4‐vinylbenzyl chloride (3.05 g, 0.02 mol) in diethyl ether (30 mL) at room temperature for 48 h under an argon atmosphere. The solvent was removed under dynamic vacuum, and the crude product was washed three times with diethyl ether to obtain [MVBIm][Cl]. ^1^H NMR (400 MHz, D_2_O, δ): 8.732 (s, 1H; Im H), 7.561 (m, 2H; Im H), 7.456 (m, 2H; Ar H), 7.386 (m, 2H; Ar H), 6.806 (m, 1H; CH), 5.882 (d, 1H; CH_2_), 5.496 (d, 1H; CH_2_), 5.367 (s, 2H; N‐CH_2_), 3.873 (s, 3H; N‐CH_3_) (see Figure S13a in the Supporting Information).

*Synthesis of 1,2‐Dimethyl‐3‐(4‐Vinylbenzyl)Imidazolium Chloride ([DMVBIm][Cl])*: [DMVBIm][Cl] was synthesized by stirring a mixture containing 1,2‐methylimidazole (1.92 g, 0.02 mol) and 4‐vinylbenzyl chloride (3.05 g, 0.02 mol) in diethyl ether (30 mL) at room temperature for 48 h under an argon atmosphere. The solvent was removed under dynamic vacuum, and the crude product was washed three times with diethyl ether to obtain [DMVBIm][Cl]. ^1^H NMR (400 MHz, D_2_O, δ): 7.490 (d, 2H; Im H), 7.317 (s, 2H; Ar H), 7.230 (d, 2H; Ar H), 6.752 (m, 1H; CH), 5.832(d, 1H; CH_2_), 5.323 (m, 3H; CH_2_), 5.281 (m, 2H; N‐CH_2_), 3.736 (s, 3H; N‐CH_3_), 2.518 (s, 3H; CH_3_) (see Figure S13b in the Supporting Information).

*Synthesis of (4‐Vinylbenzyl)Pyridinium Chloride ([VBPy][Cl])*: [VBPy][Cl] was synthesized by stirring a mixture containing pyridine (1.58 g, 0.02 mol) and 4‐vinylbenzyl chloride (3.05 g, 0.02 mol) in diethyl ether (30 mL) at room temperature for 48 h under an argon atmosphere. The solvent was removed under dynamic vacuum, and the crude product was washed three times with diethyl ether to obtain [VBPy][Cl]. ^1^H NMR (400 MHz, D_2_O, δ): 8.851 (d, 2H; Py H), 8.501 (t, 1H; Py H), 8.009 (t, 2H; Py H), 7.515 (d, 2H; Ar H), 7.390 (d, 2H; Ar H), 6.740 (q, 1H; CH), 5.841 (d, 1H; CH_2_), 5.745 (s, 2H; N‐CH_2_), 5.330 (d, 1H; CH) (see Figure S13c in the Supporting Information).

*Synthesis of 1‐(4‐Vinylbenzyl)‐1‐Methylpyrrolidinium Chloride ([VBMPL][Cl])*: [VBMPL][Cl] was synthesized by stirring a mixture containing 1‐methypyrroldine (1.70 g, 0.02 mol) and 4‐vinylbenzyl chloride (3.05 g, 0.02 mol) in diethyl ether (30 mL) at room temperature for 48 h under an argon atmosphere. The solvent was removed under dynamic vacuum, and the crude product was washed three times with diethyl ether to obtain [VBMPL][Cl]. ^1^H NMR (400 MHz, D_2_O, δ): 7.593 (d, 2H; Ar H), 7.500 (d,2H; Ar H), 6.806 (q, 1H; CH), 5.919 (d, 1H; CH_2_), 5.389 (d, 1H; CH_2_), 4.472 (s, 2H; N‐CH_2_), 3.686‐3.496 (d, 4H; N‐CH_2_), 2.937 (s, 3H; N‐CH_3_), 2.225 (s, 4H; CH_2_) (see Figure S13d in the Supporting Information).

*Synthesis of (4‐Vinylbenyl)Trimethylphosphonium Chloride ([VBTMP][Cl])*: [VBTMP][Cl] was synthesized by stirring a mixture containing trimethylphosphine tetrahydrofuran solution (10 mL, 0.01 mol) and 4‐vinylbenzyl chloride (1.53 g, 0.01 mol) at room temperature for 48 h under an argon atmosphere. The solvent was removed under dynamic vacuum, and the crude product was washed three times with diethyl ether to obtain [VBTMP][Cl]. ^1^H NMR (400 MHz, D_2_O, δ): 7.530 (d, 2H; Ar H), 7.281 (d, 2H; Ar H), 6.777 (q, 1H; CH), 5.856 (d, 1H; CH_2_), 5.327 (d, 1H; CH_2_), 3.667 (s, 2H; P‐CH_2_), 1.789 (s, 9H; P‐CH_3_) (see Figure S13e in the Supporting Information).

*Synthesis of Cationic Polymers*: The cationic polymers were synthesized via free radical polymerization of cationic monomer using azobis(isobutyronitrile) (AIBN) as the initiator in DMSO. For example, poly(1‐methyl‐3‐(4‐vinylbenzyl) imidazolium chloride) ([PMVBIm][Cl]) was synthesized by stirring a mixture containing [MVBIm][Cl] and 1 wt% AIBN dissolved in DMSO at 60 °C for 8 h under a nitrogen atmosphere. The product was precipitated with chloroform and washed three times with chloroform and then dried under vacuum at 60 °C. Poly(1,2‐dimethyl‐3‐(4‐vinylbenzyl)imidazolium chloride) ([PDMVBIm][Cl]), poly((4‐vinylbenyl) trimethylphosphonium chloride) ([PVBTMP][Cl]), poly((4‐vinylbenzyl)pyridinium chloride) ([PVBPy][Cl]), poly(1‐(4‐vinylbenzyl)‐1‐methylpyrrolidinium chloride) ([PVBMPL][Cl]) and poly((4‐vinylbenyl) trimethylammonium chloride) ([PVBTMA][Cl]) were synthesized following the same procedure (NMR spectra, see Figure S14, Supporting Information).

*Characterization*: ^1^H NMR spectra were measured using a Varian spectrometer at 400 MHz to evaluate the alkaline stability of cations and cationic polymers.

*Alkaline Stability Measurements*: Alkaline stability of organic cations was studied in a variety of KOH solutions $\begin{array}{l} (\mathrm{including}\;\;\;D_{2}O,\;\;V_{D_{2}O}/V_{{\mathrm{CD}}_{3}\mathrm{OD}}=\;\;3:1,\;\;\;V_{D_{2}O}/V_{{\mathrm{CD}}_{3}\mathrm{OD}}=\;\;\;1:1,\;\;V_{D_{2}O}/V_{{\mathrm{CD}}_{3}\mathrm{OD}}\;\;=\;\;\;1:3,\;\\ {\mathrm{CD}}_{3}\mathrm{OD},\;\;V_{D_{2}O}/V_{{\mathrm{CD}}_{3}{\mathrm{CD}}_{2}\mathrm{OD}}\;\;\;=\;\;1:1,\;\;V_{D_{2}O}/V_{{\mathrm{CD}}_{3}{\mathrm{CD}}_{2}\mathrm{OD}}=1:3\;\;\mathrm{and}\;\;V_{\mathrm{DMSO}}/V_{D_{2}O}=\;\;1:1)\;\;.\;\; \end{array}$ All the test solutions were placed in polypropylene jars and kept at 80 °C for various times. Aliquots were sampled and immediately transferred into a standard NMR tube for ^1^H NMR characterization. The alkaline degradation degree of the cationic polymers was determined under the same way.

*Computational Details and Analysis*: Theoretical analysis of various organic cations was evaluated in a variety of fuels, including water, DMSO, methanol and ethanol, by Dmol^62^ density functional code^63^ available in Materials Studio (Version 7.0). The GGA‐BLYP functional^64^, ^65^ and DNP basis set was used for all the calculations. The geometries of the molecules were fully optimized with a self‐consistent field (SCF) convergence value of 10^−6^ Ha. The convergence criteria were 5 × 10^−6^ Ha for energy, 0.005 Å for displacement, and 0.001 Ha Å^−1^ for gradient during the geometry optimization. The dielectric constant of solvents water (ε = 78.54), DMSO (ε = 46.70), methanol (ε = 32.63) and ethanol (ε = 24.30) was used for the calculations.

To ensure the frequencies are all normal, a frequency analysis was applied to all the optimized single molecules. In searching TS structures, a complete linear synchronous transit/quadratic synchronous transit (LST/QST) method was adopted.^66^ Along the reaction coordinate, only one imaginary frequency was observed in all the obtained TS structures. The nudged elastic band (NEB) available in the following TS confirmation is carried out to ensure that the TS geometries directly connect the corresponding reactants and products.^67^

## Conflict of Interest

The authors declare no conflict of interest.

## Supporting information

Supplementary
