# Supplementary material for: The Alkaline Stability of Anion Exchange Membrane for Fuel Cell Applications: The Effects of Alkaline Media
Source: Adv Sci (Weinh). 2018 Jun 4;5(8):1800065. doi: 10.1002/advs.201800065 (PMC6097010; doi:10.1002/advs.201800065)
Supplement: Supplementary file 1 — Supplementary [file ADVS-5-1800065-s001.pdf]

Copyright WILEY-VCH Verlag GmbH & Co. KGaA, 69469 Weinheim, Germany, 2018.

ADVANCED SCIENCE
Open Access

Supporting Information

for *Adv. Sci.,* DOI: 10.1002/advs.201800065

The Alkaline Stability of Anion Exchange Membrane for Fuel Cell Applications: The Effects of Alkaline Media

*Zhe Sun, Ji Pan, Jiangna Guo, and Feng Yan**

Copyright WILEY-VCH Verlag GmbH & Co. KGaA, 69469 Weinheim, Germany, 2016.

Supporting Information

The Alkaline Stability of Anion Exchange Membrane for Fuel Cell Applications: the Effects of Alkaline Media

*Zhe Sun, Ji Pan, Jiangna Guo and Feng Yan**

State and Local Joint Engineering Laboratory for Novel Functional Polymeric Materials, Department of Polymer Science and Engineering, College of Chemistry, Chemical Engineering and Materials Science, Soochow University, Suzhou, 215123, China.

E-mail: fyan@suda.edu.cn

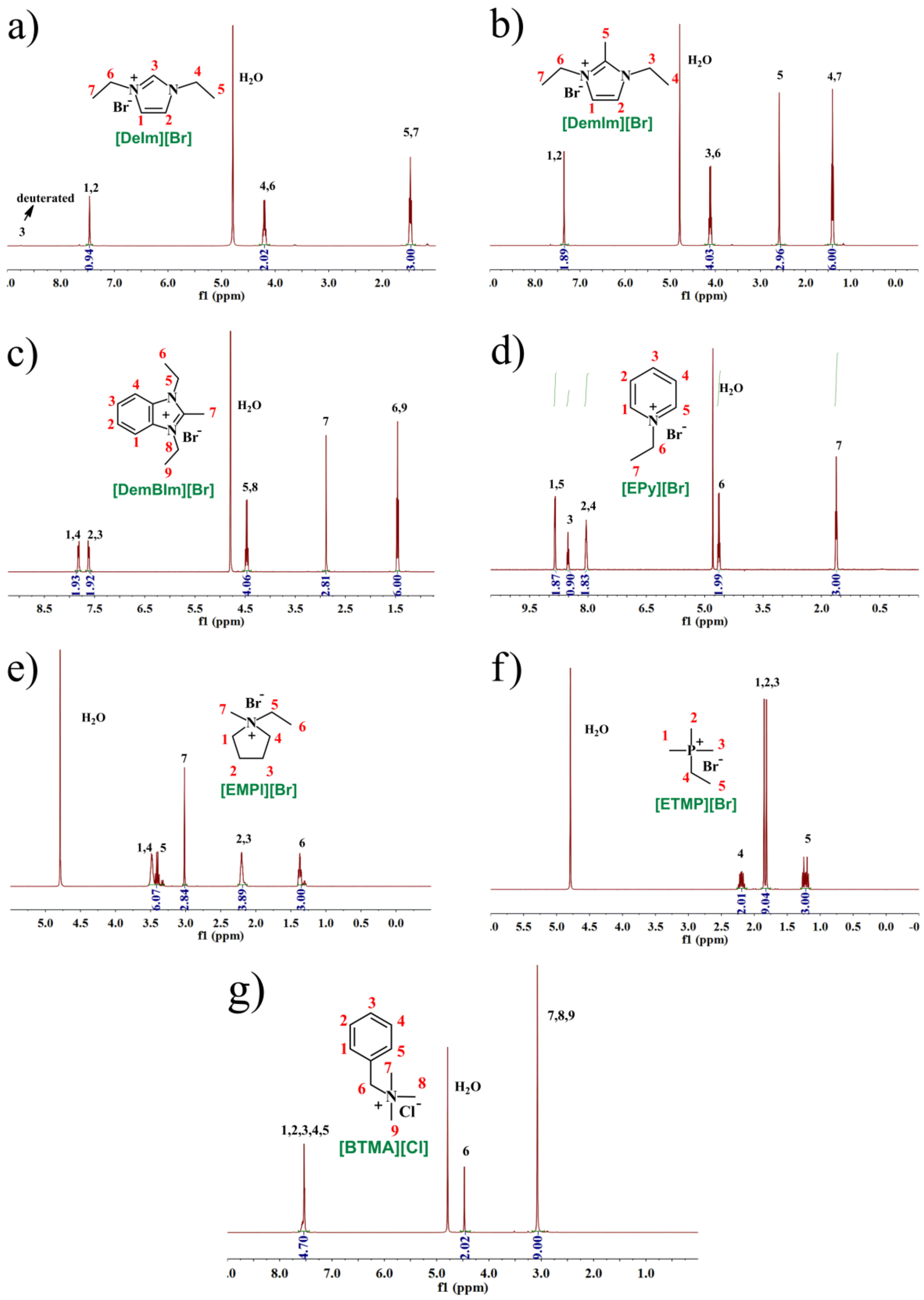

Figure S1. ^{1}H NMR spectra for various organic cations studied in this work including: (a) [DeIm][Br], (b) [DemIm][Br], (c) [DemBIm][Br], (d) [EPy][Br], (e) [EMPl][Br], (f) [ETMP][Br] and (g) [BTMA][Cl].

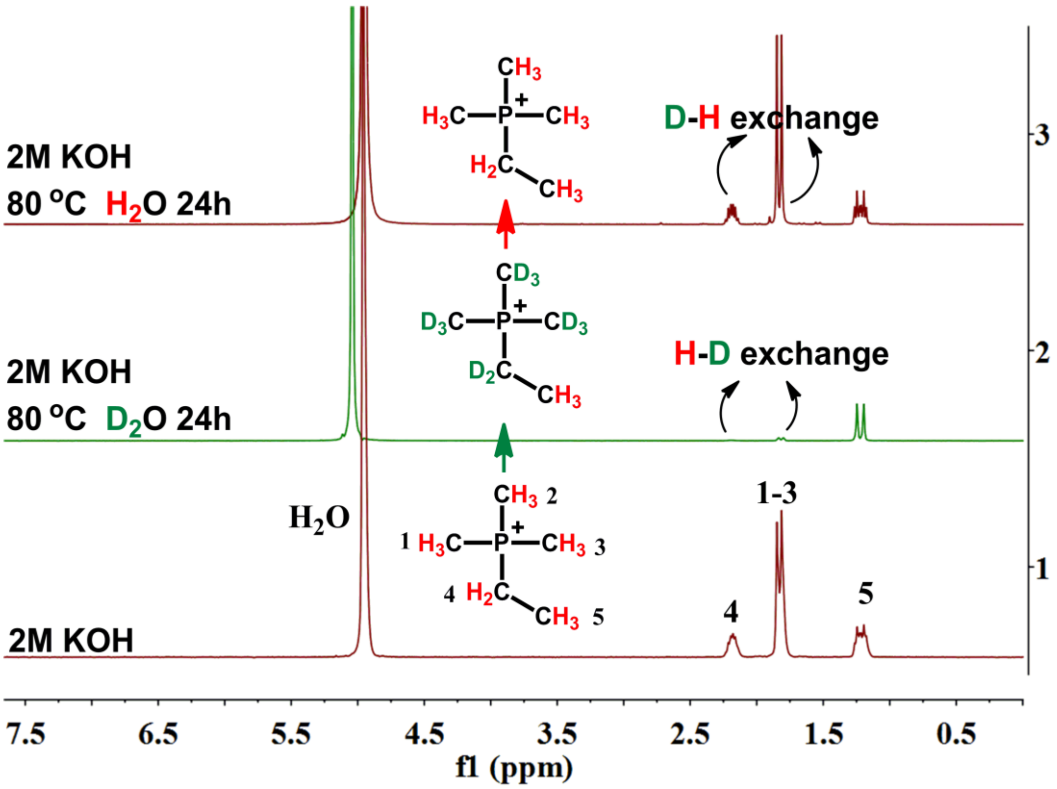

Figure S2. ^{1}H NMR spectra of $[ETMP]^+$ in 2 M KOH/D_2O at 80 ^oC for 24 h, before and after a second storage period in 2 M KOH/H_2O for 24 h at 80 ^oC. The green and red arrows indicate the disappearance and reappearance of α-C protons after the interconversion of hydrogen and deuterium, respectively.

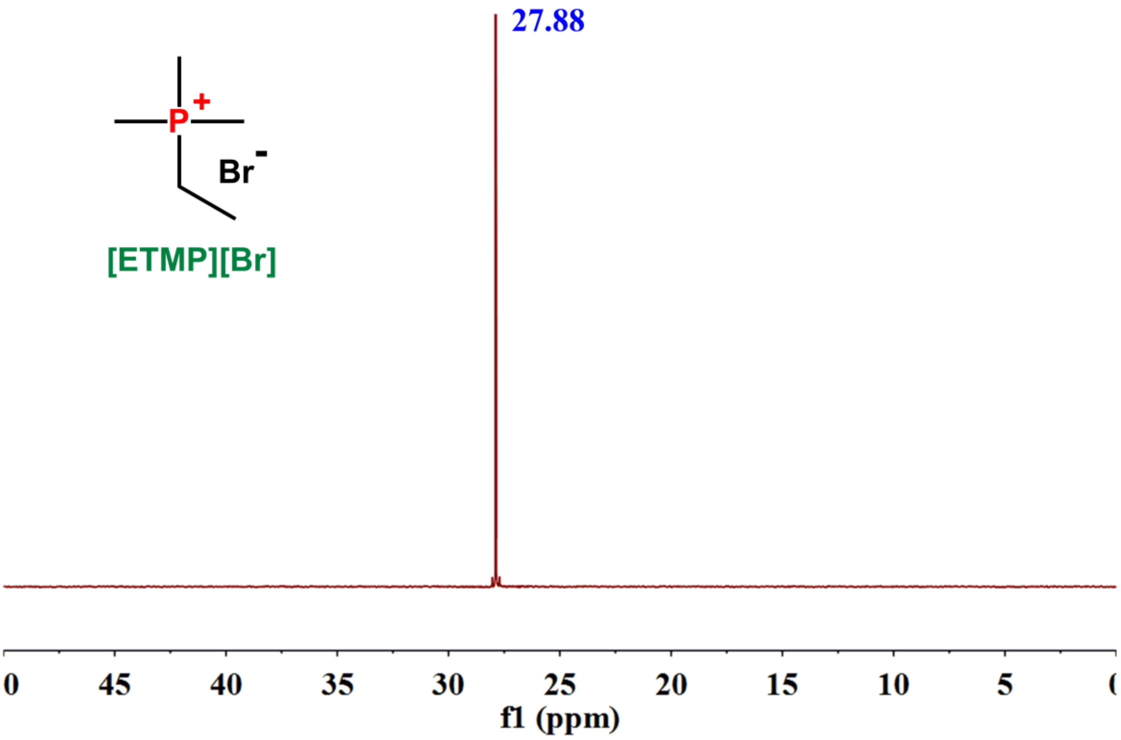

Figure S3. ^{31}P NMR spectra for [ETMP][Br].

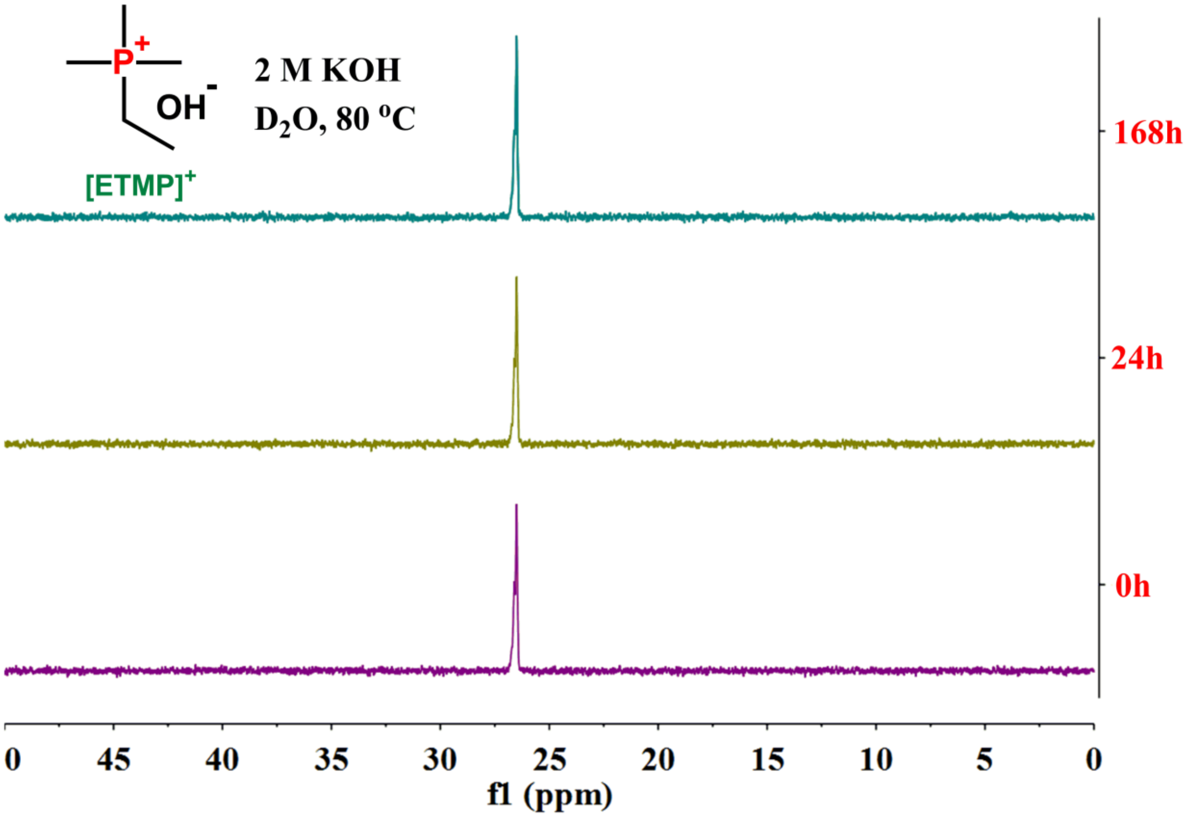

Figure S4. ^{31}P NMR spectra for $[ETMP]^+$ in 2 M KOH ([KOH]/[cation] = 15/1, molar ratio) D_2O solution at 80 ℃ for 0, 24 and 168h, respectively.

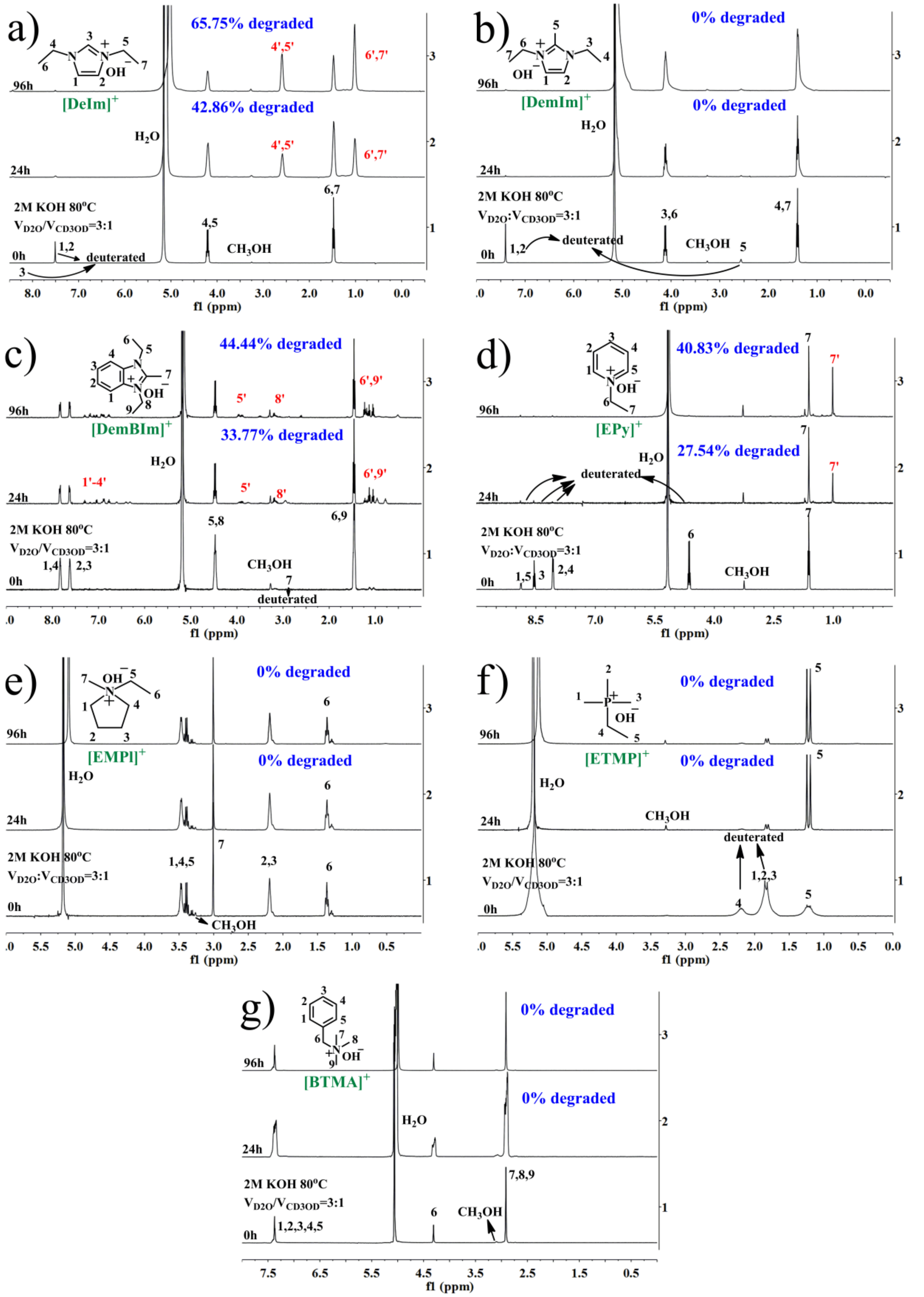

Figure S5. ^{1}H NMR spectra for (a) $[DeIm]^+$, (b) $[DemIm]^+$, (c) $[DemBIm]^+$, (d) $[EPy]^+$, (e) $[EMPl]^+$, (f) $[ETMP]^+$ and (g) $[BTMA]^+$ in 2 M KOH ([KOH]/[cation] = 15/1, molar ratio) mixed solution (V_{D_2O}/V_{CD_3OD}=3:1) at 80 ℃ for 24 and 96h, respectively.

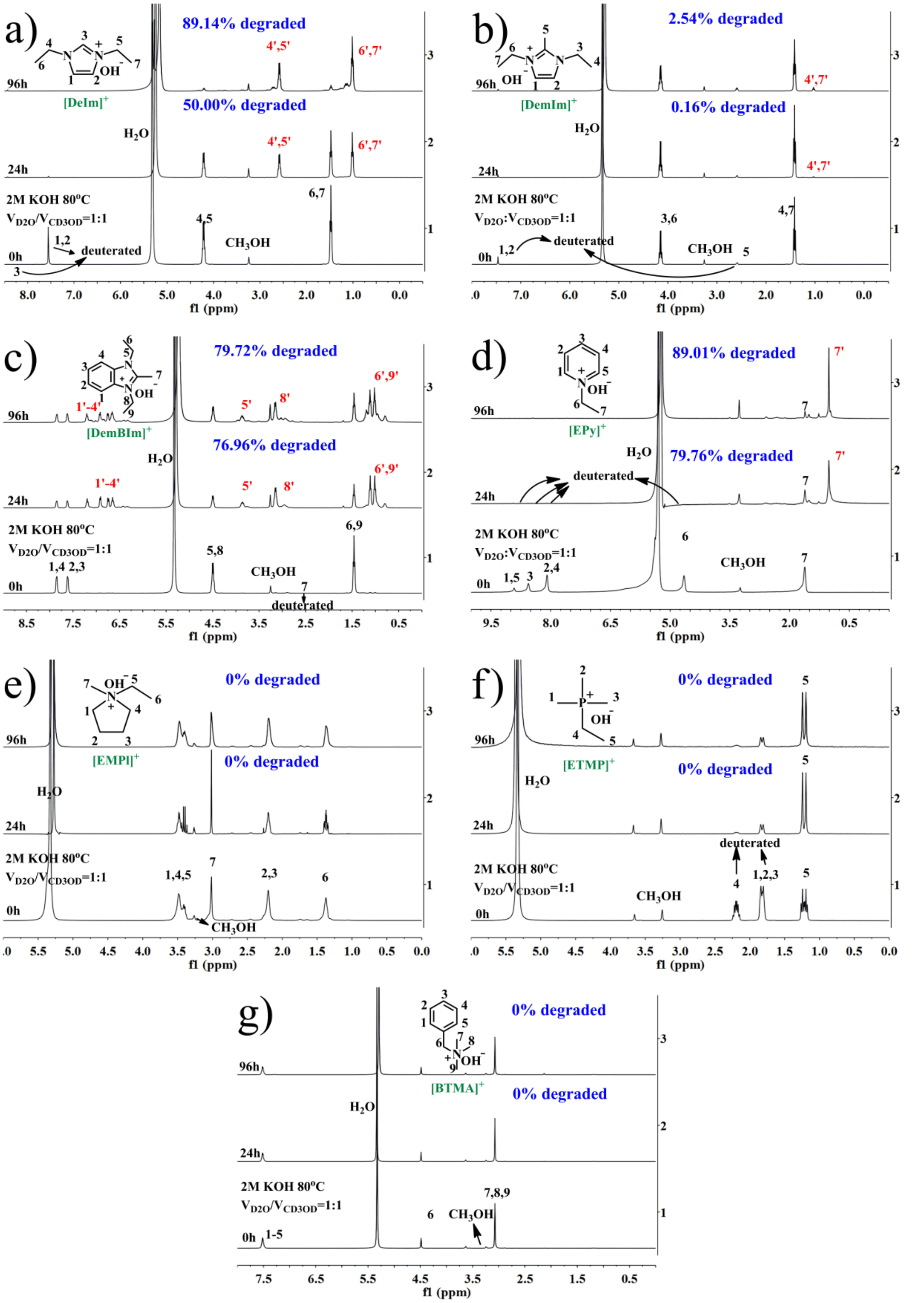

Figure S6. ^{1}H NMR spectra for (a) [DeIm]+, (b) [DemIm]+, (c) [DemBIm]+, (d) [EPy]+, (e) [EMPl]+, (f) [ETMP]+ and (g) [BTMA]+ in 2 M KOH ([KOH]/[cation] = 15/1, molar ratio) mixed solution (V_{D_2O}/V_{CD_3OD}=1:1) at 80 ℃ for 24 and 96h, respectively.

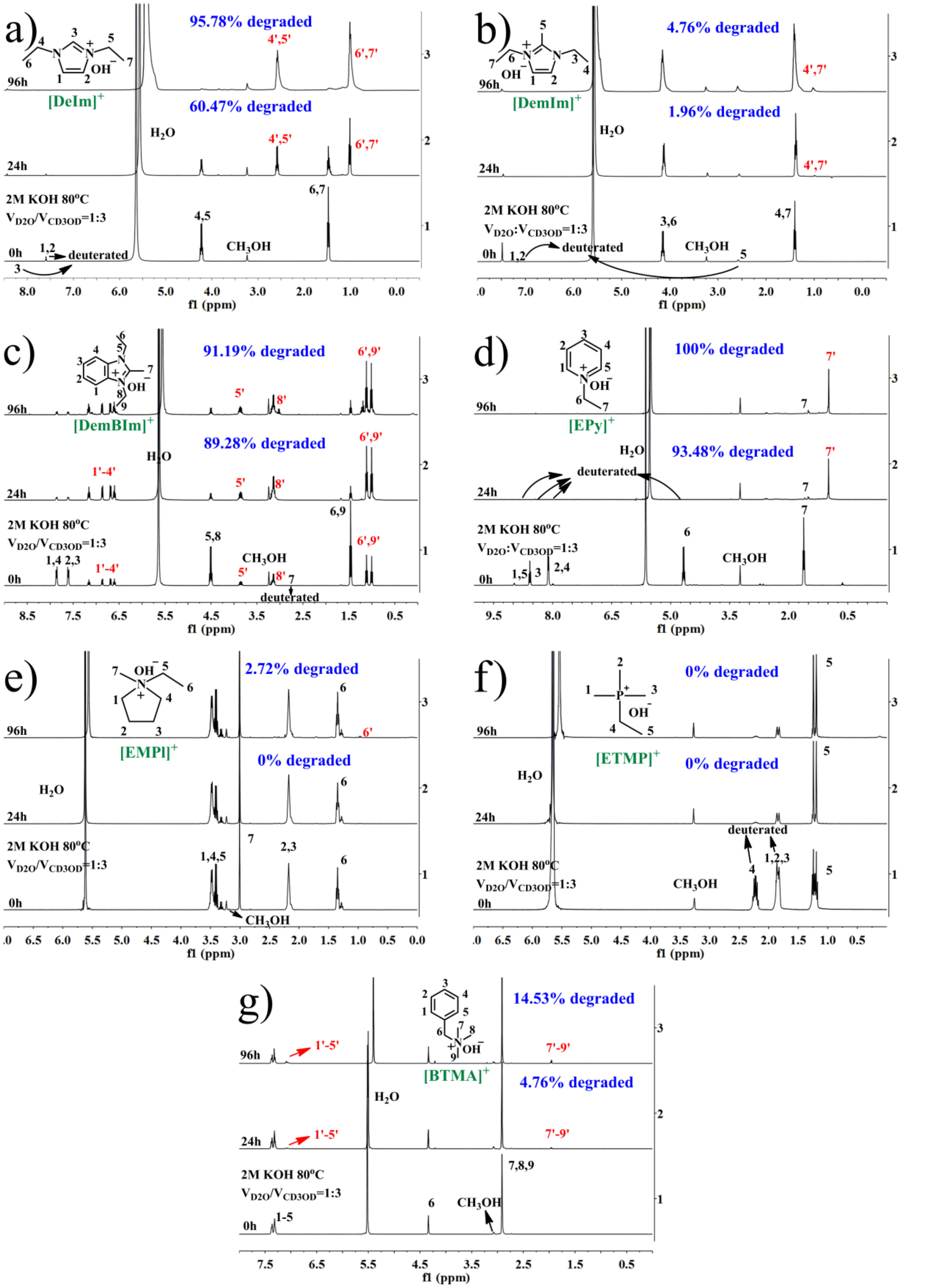

Figure S7. ^{1}H NMR spectra for (a) $[DeIm]^+$, (b) $[DemIm]^+$, (c) $[DemBIm]^+$, (d) $[EPy]^+$, (e) $[EMPl]^+$, (f) $[ETMP]^+$ and (g) $[BTMA]^+$ in 2 M KOH ([KOH]/[cation] = 15/1, molar ratio) mixed solution (V_{D_2O}/V_{CD_3OD}=1:3) at 80 ℃ for 24 and 96h, respectively.

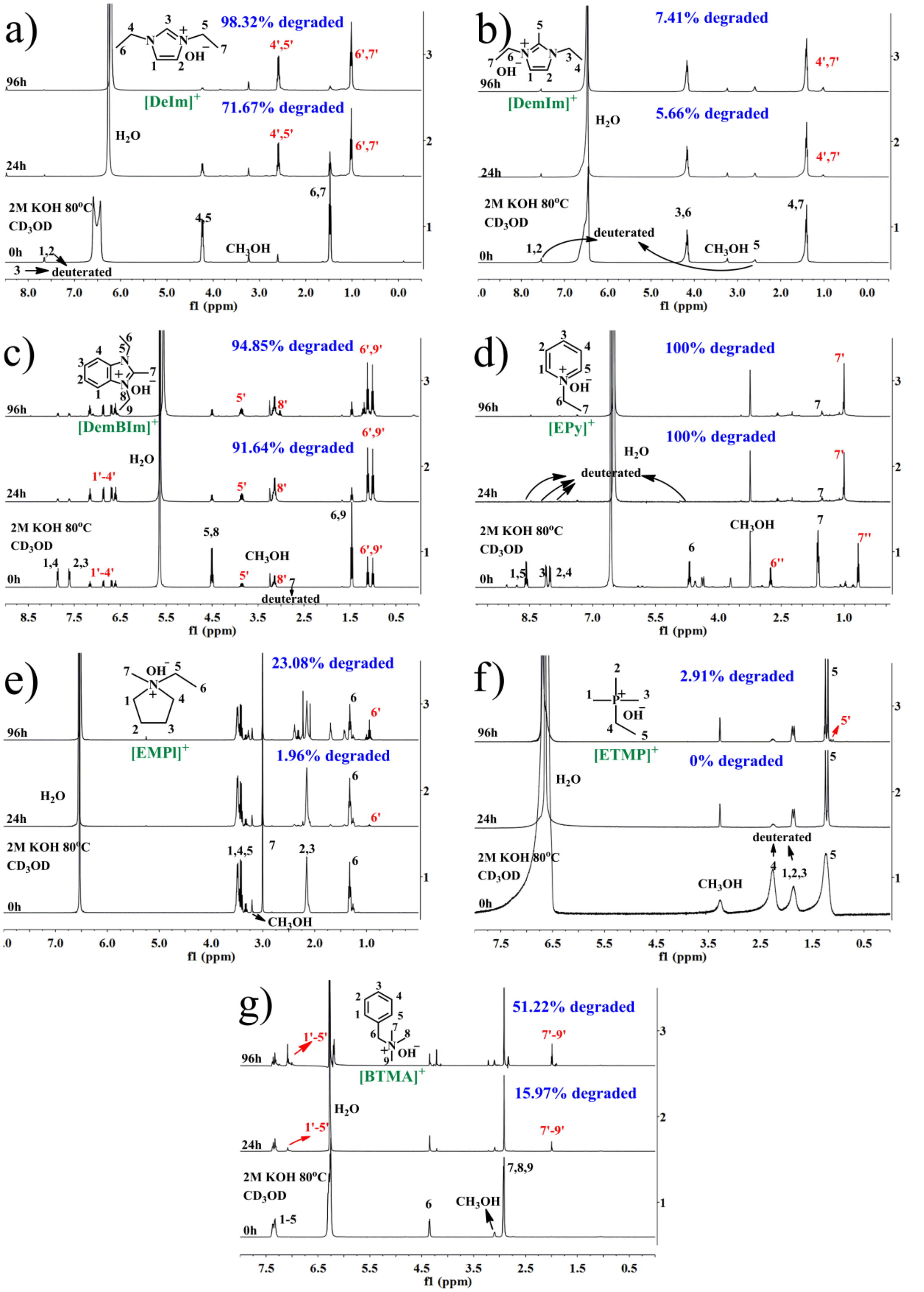

Figure S8. ^{1}H NMR spectra for (a) $[DeIm]^+$, (b) $[DemIm]^+$, (c) $[DemBIm]^+$, (d) $[EPy]^+$, (e) $[EMPl]^+$, (f) $[ETMP]^+$ and (g) $[BTMA]^+$ in 2 M KOH ([KOH]/[cation] = 15/1, molar ratio) CD_3OD solution at 80 ℃ for 24 and 96h, respectively.

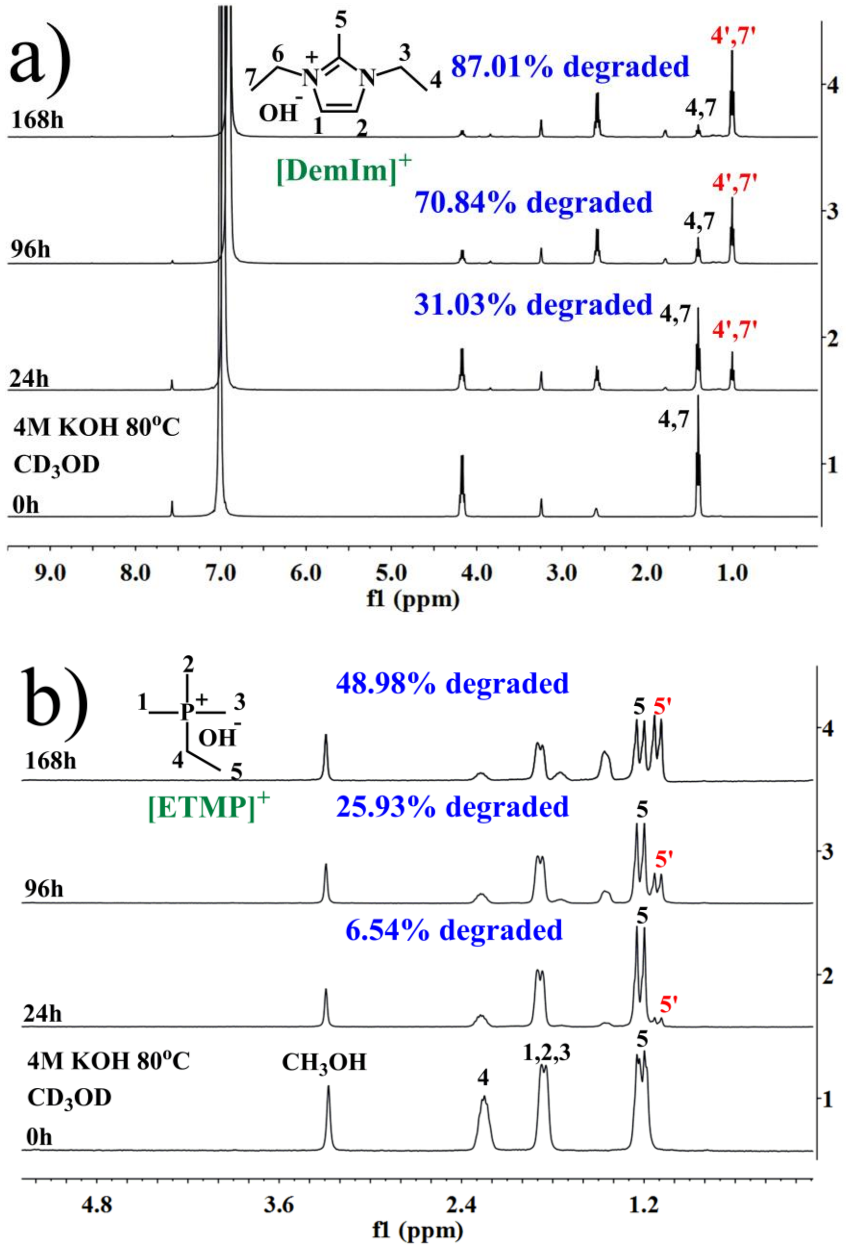

Figure S9. ^{1}H NMR spectra for (a) $[DemIm]^+$, (b) $[ETMP]^+$ in 4 M KOH CD_3OD solutions ([KOH]/[cation] = 15/1, molar ratio) at 80 ℃ for 24, 96 and 168h, respectively.

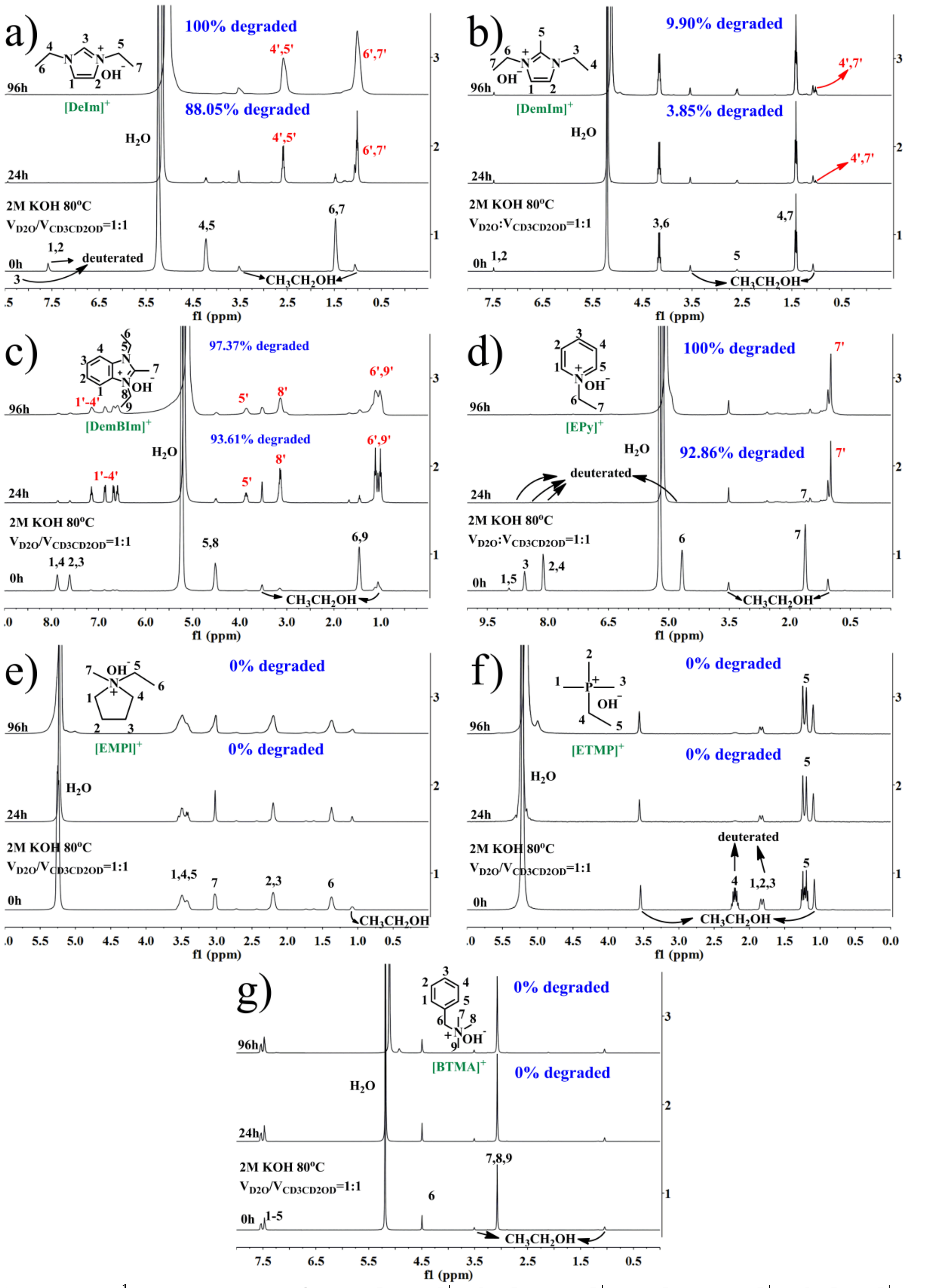

Figure S10. ^{1}H NMR spectra for (a) $[DeIm]^+$, (b) $[DemIm]^+$, (c) $[DemBIm]^+$, (d) $[EPy]^+$, (e) $[EMPl]^+$, (f) $[ETMP]^+$ and (g) $[BTMA]^+$ in 2 M KOH ([KOH]/[cation] = 15/1, molar ratio) mixed solution ($V_{D_2O}/V_{CD_3CD_2OD}$=1:1) at 80 ℃ for 24 and 96h, respectively.

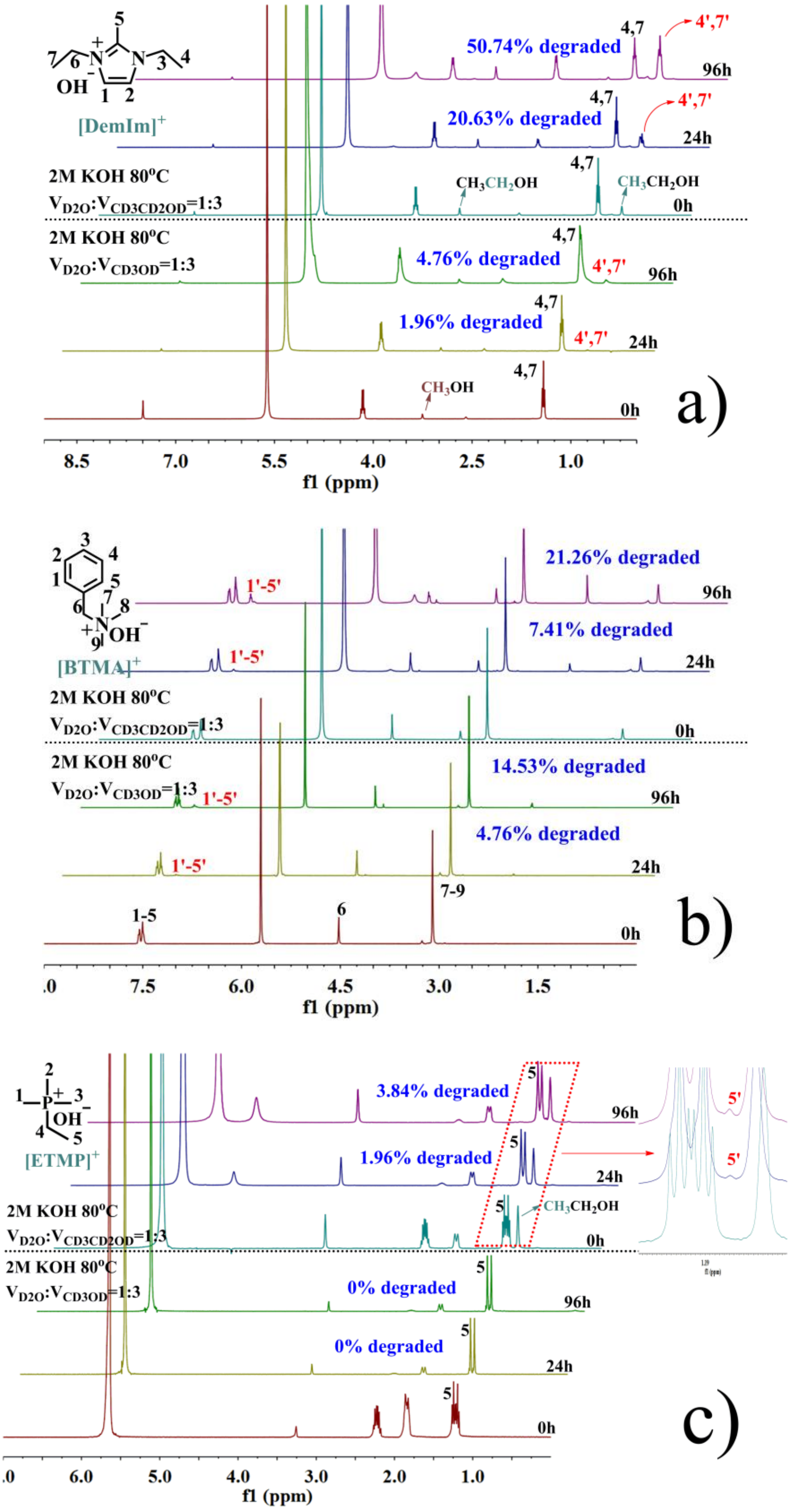

Figure S11. ^{1}H NMR spectra for (a) $[DemIm]^{+}$, (b) $[BTMA]^{+}$ and (c) $[ETMP]^{+}$ in 2 M KOH ([KOH]/[cation] = 15/1, molar ratio) V_{D_2O}/V_{CD_3OD}=1:3 and $V_{D_2O}/V_{CD_3CD_2OD}$=1:3 solution at 80 ℃ for 24 and 96h, respectively.

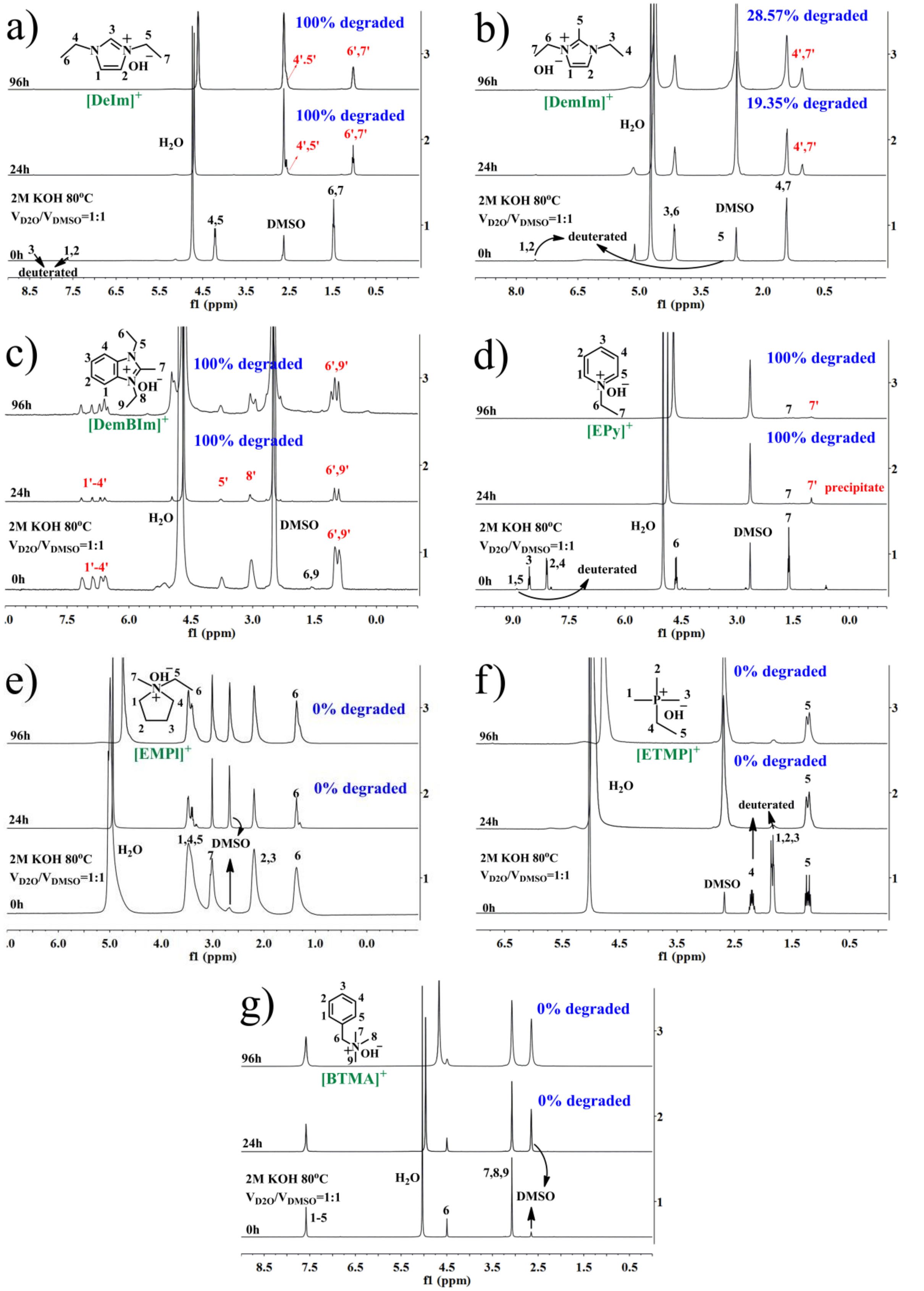

Figure S12. 1H NMR spectra for (a) $[DeIm]^{+}$, (b) $[DemIm]^{+}$, (c) $[DemBIm]^{+}$, (d) $[EPy]^{+}$, (e) $[EMPl]^{+}$, (f) $[ETMP]^{+}$ and (g) $[BTMA]^{+}$ in 2 M KOH ([KOH]/[cation] = 15/1, molar ratio) mixed solution (V_{D_2O}/V_{DMSO}=1:1) at 80 ℃ for 24 and 96h, respectively.

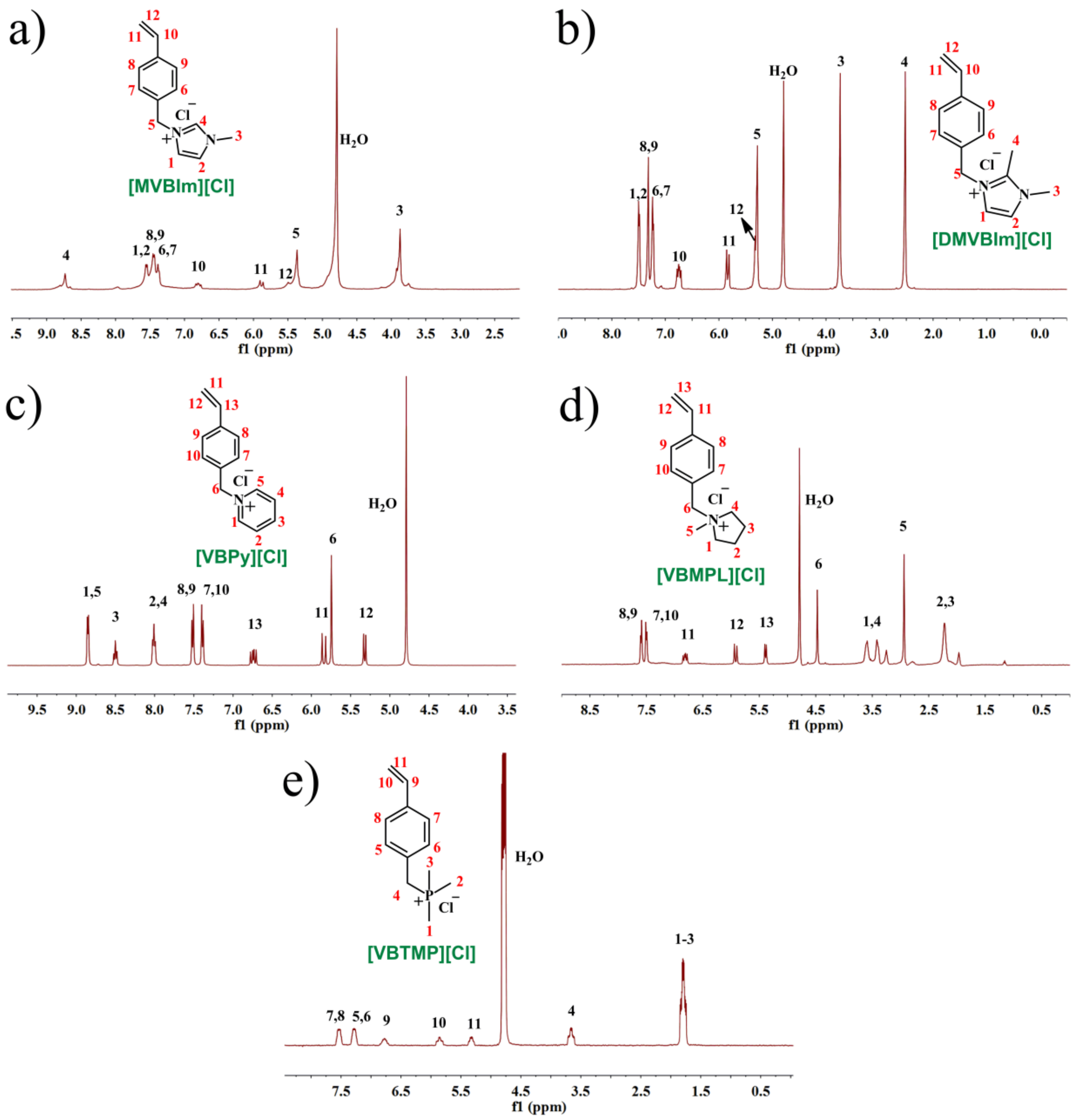

Figure S13. ^{1}H NMR spectra for the vinyl monomer synthesized in this work including: (a) [MVBIm][Cl], (b) [DMVBIm][Cl], (c) [VBPy][Cl], (d) [VBMPl][Cl] and (e) [VBTMP][Cl].

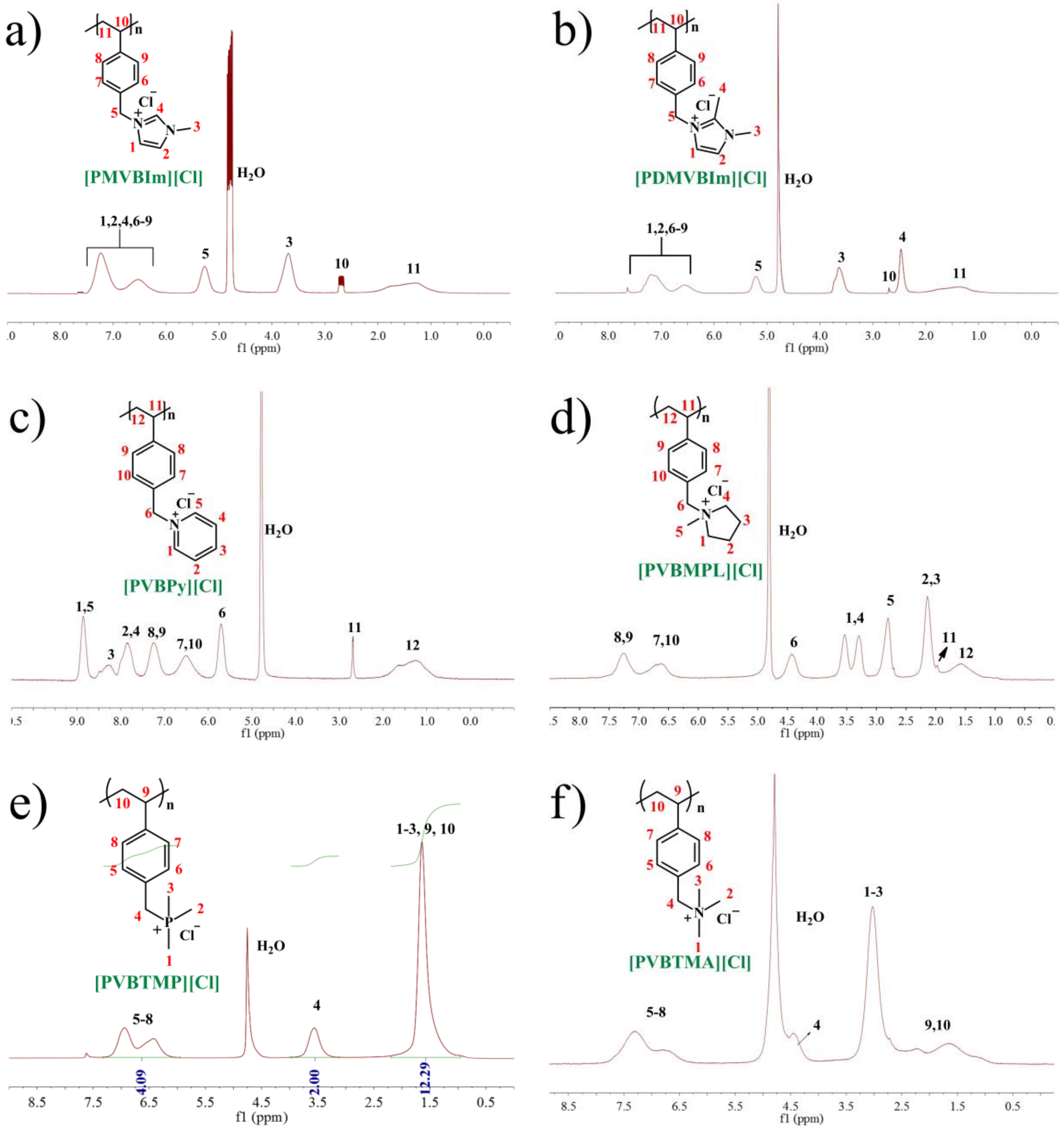

Figure S14. ^{1}H NMR spectra for various cationic polymers studied in this work including: (a) [PMVBIm][Cl], (b) [PDMVBIm][Cl], (c) [PVBPy][Cl], (d) [PVBMPl][Cl], (e) [PVBTMP][Cl] and (f) [PVBTMA][Cl].

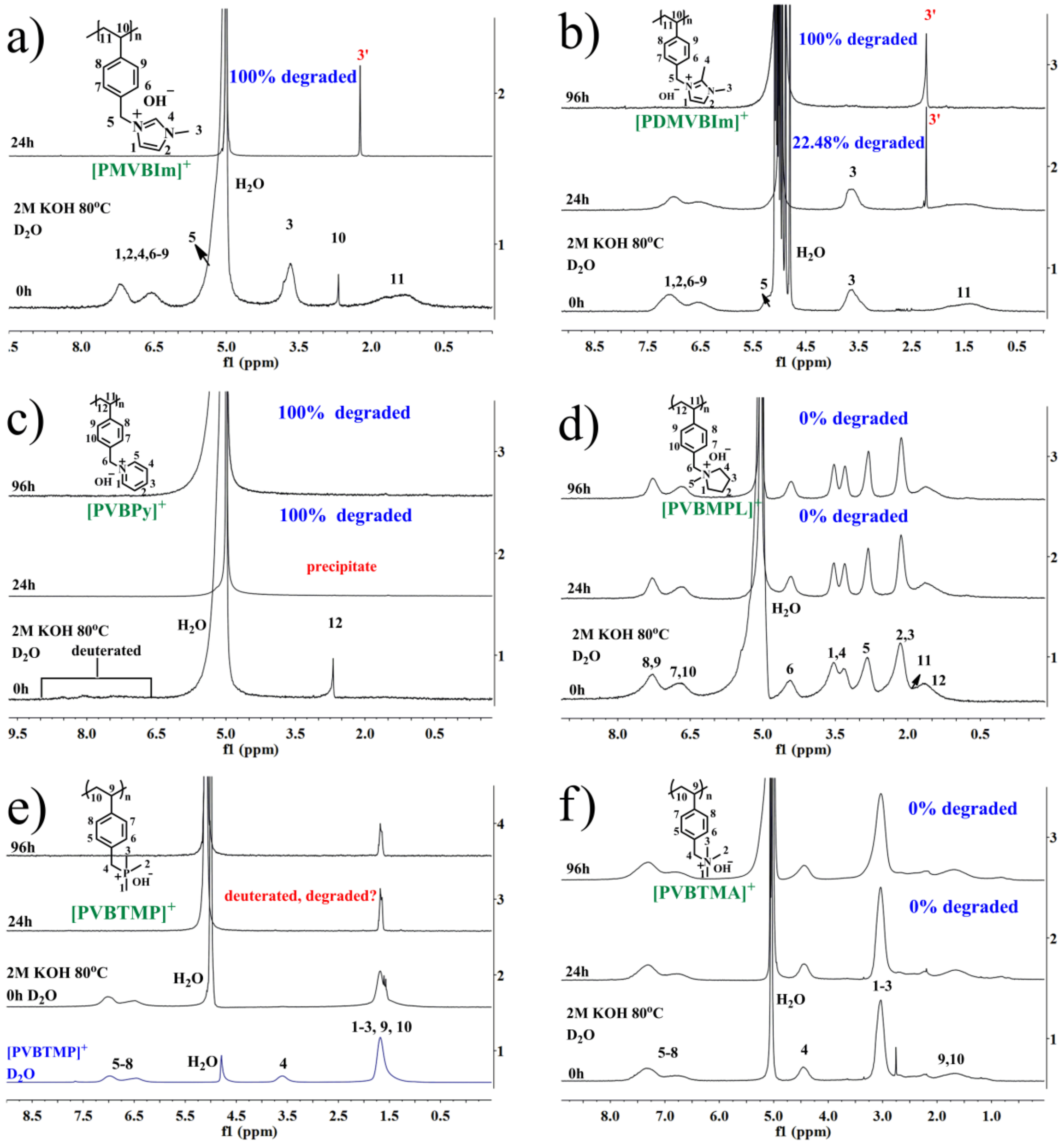

Figure S15. ^{1}H NMR spectra for (a) $[PMVBIm]^+$, (b) $[PDMVBIm]^+$, (c) $[PVBPy]^+$, (d) $[PVBMPl]^+$, (e) $[PVBTMP]^+$ and (f) $[PVBTMA]^+$ in 2 M KOH ([KOH]/[cation] = 15/1, molar ratio) D_2O solution at 80 ℃ for 24 and 96 h, respectively.

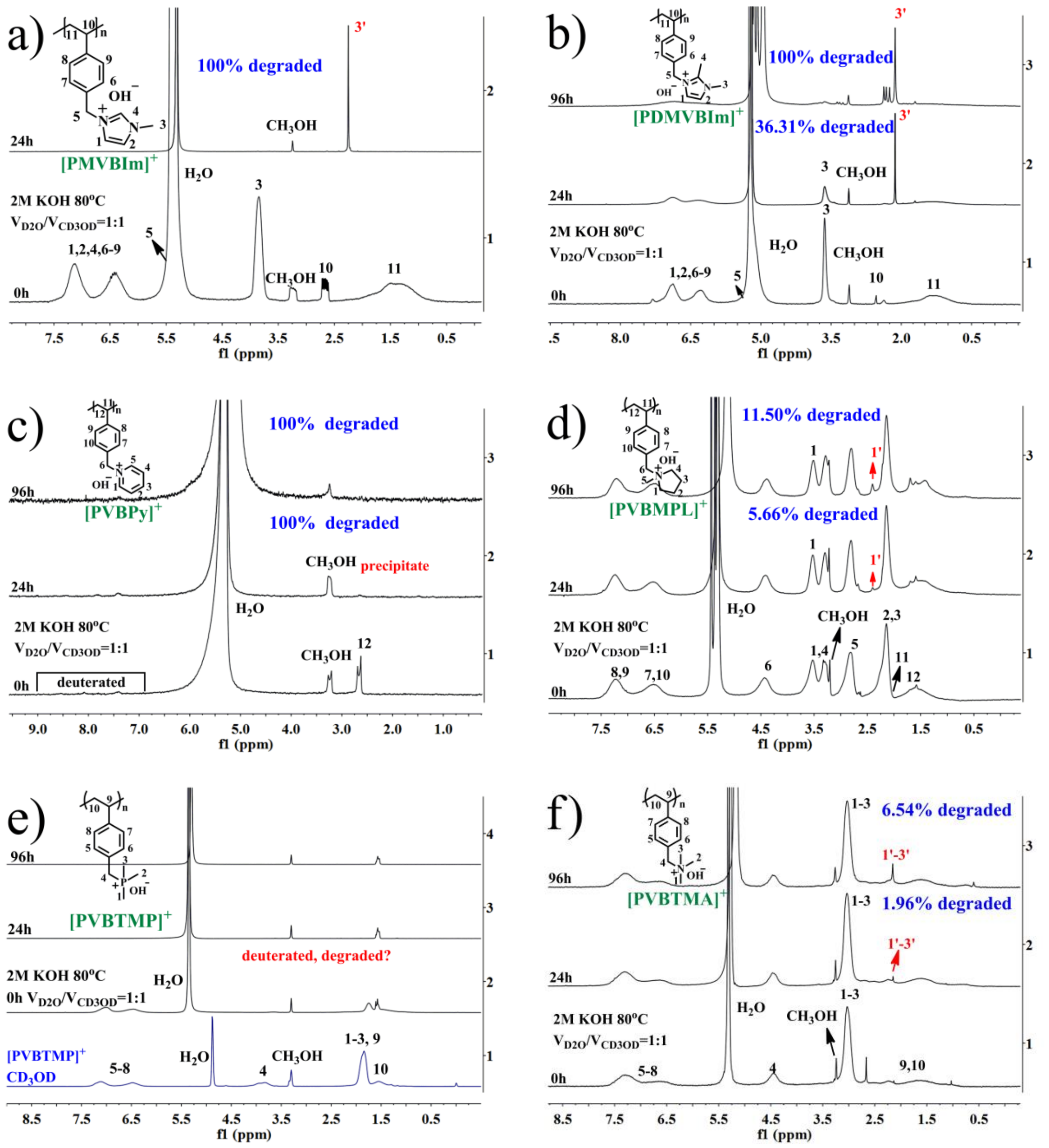

Figure S16. ^{1}H NMR spectra for (a) [PMVBIm]+, (b) [PDMVBIm]$^{+}$, (c) [PVBPy]$^{+}$, (d) [PVBMPl]$^{+}$, (e) [PVBTMP]$^{+}$ and (f) [PVBTMA]$^{+}$ in 2 M KOH ([KOH]/[cation] = 15/1, molar ratio) mixed solution (V_{D_2O}/V_{CD_3OD}=1:1) at 80 ℃ for 24 and 96 h, respectively.

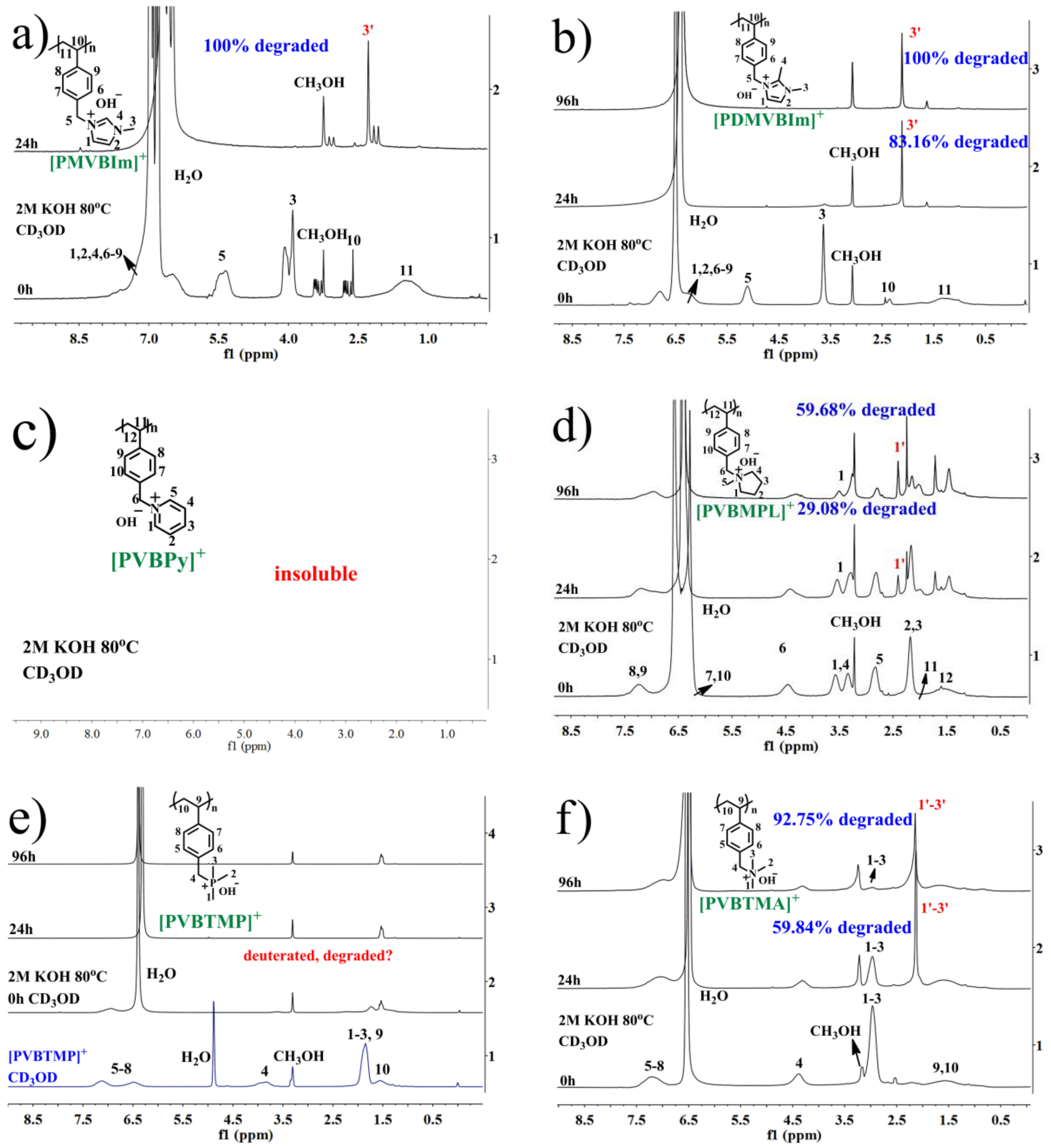

Figure 17. ^{1}H NMR spectra for (a) [PMVBIm]$^{+}$, (b) [PDMVBIm]$^{+}$, (c) [PVBPy]$^{+}$, (d) [PVBMPl]$^{+}$, (e) [PVBTMP]$^{+}$ and (f) [PVBTMA]$^{+}$ in 2 M KOH ([KOH]/[cation] = 15/1, molar ratio) CD_3OD solution at 80 ℃ for 24 and 96h, respectively.

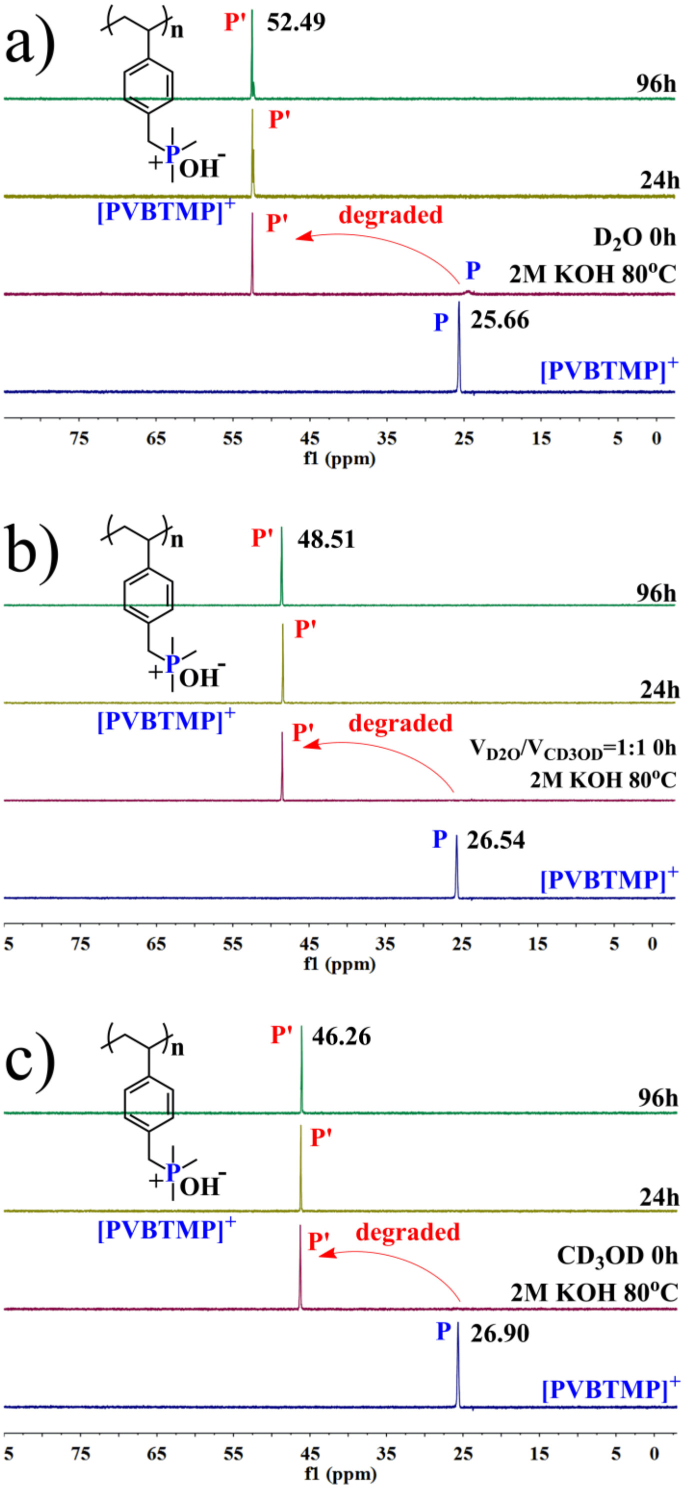

Figure S18. ^{31}P NMR spectra for $[PVBTMP]^+$ and $[PVBTMP]^+$ in 2 M KOH ([KOH]/[cation] = 15/1, molar ratio) (a) D_2O, (b) V_{D_2O}/V_{CD_3OD}=1:1 and (c) CD_3OD solutions at 80 ℃ for 0, 24 and 96h, respectively.

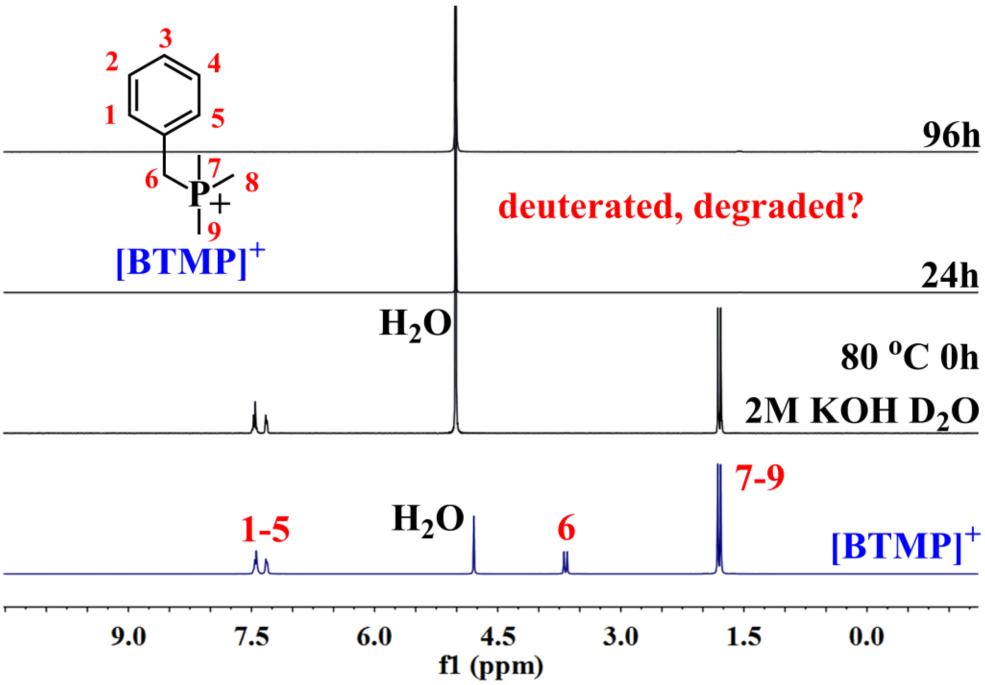

Figure S19. ^{1}H NMR spectra for $[BTMP]^+$ and $[BTMP]^+$ in 2 M KOH ([KOH]/[cation] = 15/1, molar ratio) D_2O solution at 80 ℃ for 0, 24 and 96h, respectively.

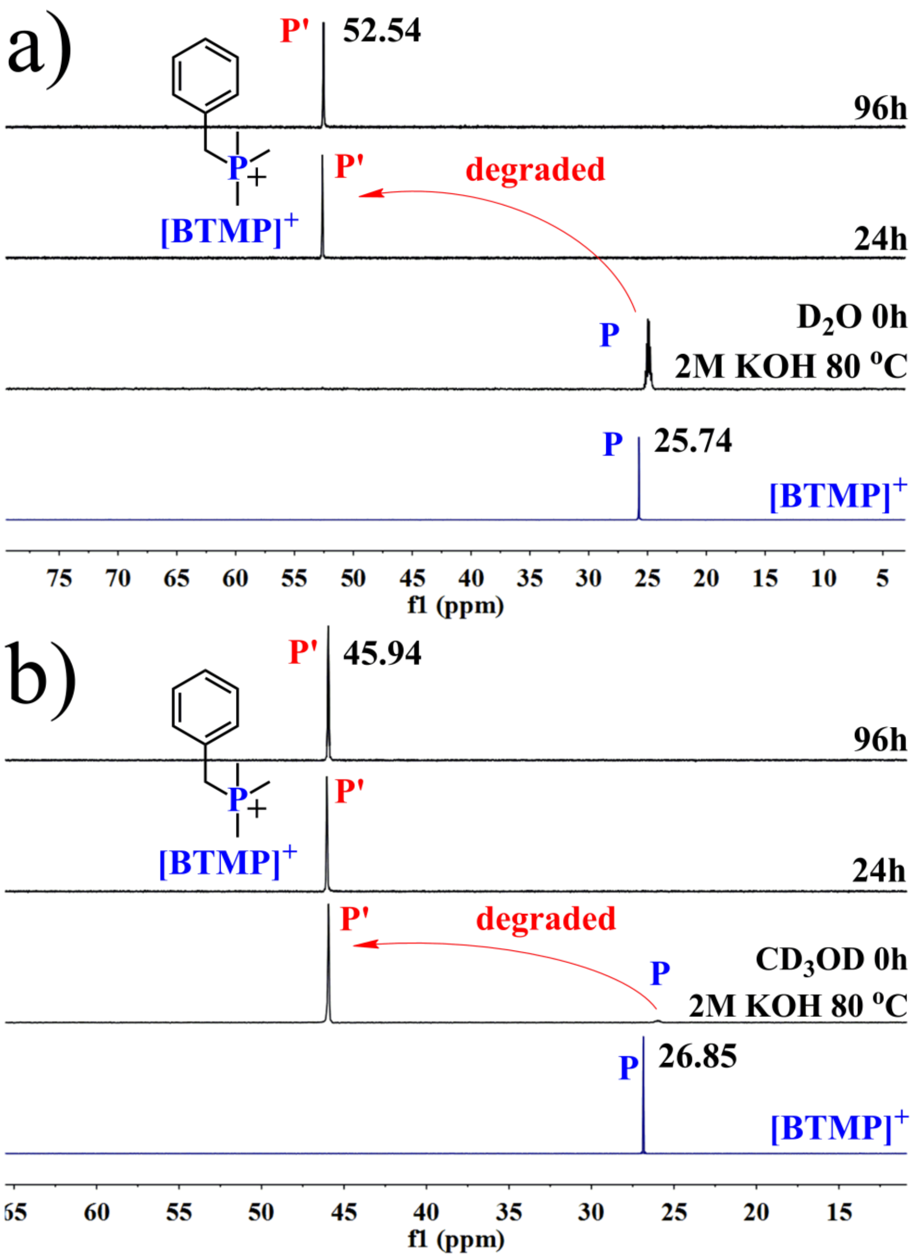

Figure S20. ^{31}P NMR spectra for $[BTMP]^+$ and $[BTMP]^+$ in 2 M KOH ([KOH]/[cation] = 15/1, molar ratio) (a) D_2O and (b) CD_3OD solutions at 80 ℃ for 0, 24 and 96h, respectively.

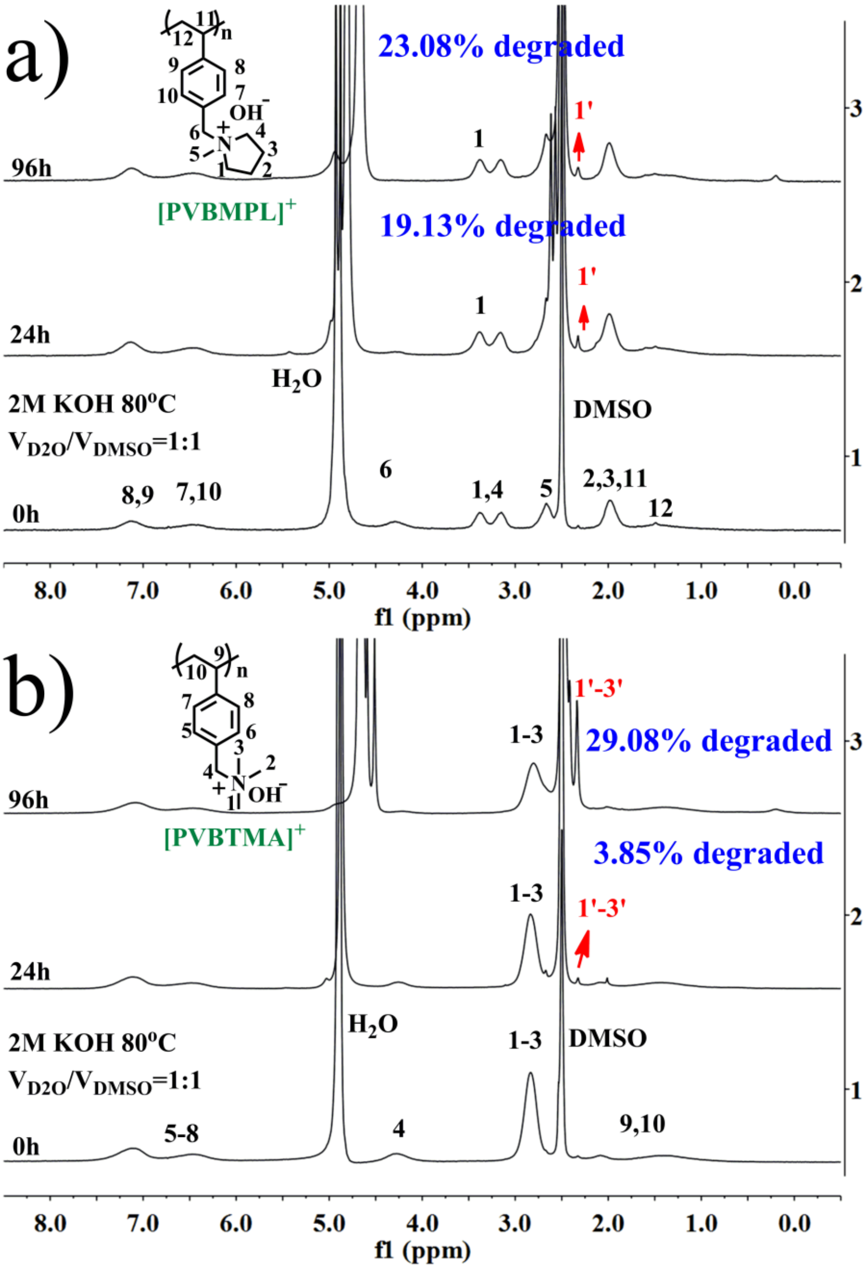

Figure S21. ^{1}H NMR spectra for (a) $[PVBMPl]^+$ and (b) $[PVBTMA]^+$ in 2 M KOH ([KOH]/[cation] = 15/1, molar ratio) mixed solution (V_{D_2O}/V_{DMSO}=1:1) at 80 ℃ for 0, 24 and 96h, respectively.

Table S1. The IEC values of cationic polymers in 2 M KOH ([KOH]/[cation] = 15/1, molar ratio) solutions at 80 ℃ for 96 h.

**Cationic Polymers**	**IEC (mmol g^{-1})**				
	**Initial value**	**D_2O (2 M KOH 96h)**	**V_{D_2O}/V_{CD_3OD}=1:1 (2 M KOH 96h)**	**CD_3OD (2 M KOH 96h)**	**V_{D_2O}/V_{DMSO}=1:1 (2 M KOH 96h)**
[PVBMPL][OH]	4.566	4.566	4.230	2.396	3.858
[PVBTMA][OH]	5.181	5.181	4.941	0.524	4.033
[PDMVBIm][OH]	4.348	0.000	0.000	0.000	\
[PMVBIm][OH]	4.630	0.000	0.000	0.000	\
[PVBPy][OH]	4.695	0.000	0.000	0.000	\
[PVBTMP][OH]	4.762	0.000	0.000	0.000	\
